# The nonlinear fast diffusion equation on smooth metric measure spaces: Hamilton-Souplet-Zhang estimates and a Ricci-Perelman super flow

**DOI:** 10.1007/s00526-025-02938-2

**Published:** 2025-02-06

**Authors:** Ali Taheri, Vahideh Vahidifar

**Affiliations:** School of Mathematical and Physical Sciences, University of Sussex, Falmer, Brighton, UK

**Keywords:** 53E20, 58J60, 58J35, 60J60

## Abstract

This article presents new gradient estimates for positive solutions to the nonlinear fast diffusion equation on smooth metric measure spaces, involving the *f*-Laplacian. The gradient estimates of interest are of Hamilton-Souplet-Zhang or elliptic type and are established using different methods and techniques. Various implications, notably to parabolic Liouville type results and characterisation of ancient solutions are given. The problem is considered in the general setting where the metric and potential evolve under a super flow involving the Bakry-Émery *m*-Ricci curvature tensor. The curious interplay between geometry, nonlinearity, and evolution – and their intricate roles in the estimates and the maximum exponent range of fast diffusion – is at the core of the investigation.

## Introduction

Our principal aim in this paper is to study the nonlinear fast diffusion equation in the framework of smooth metric measure spaces (hereafter denoted SMMS for brevity). There are many motivations and reasons for this study. Some, in particular include, understanding the analytic and dynamic behaviour of solutions, the probabilistic and stochastic features of diffusion on SMMS, the role of geometry and curvature on the one hand and nonlinearity on the other in forming the behaviour and properties of solutions, and last but not least, functional and geometric inequalities on SMMS and their close ties with the fast diffusion equation that has seen a huge revival of interest and progress in recent years (*see* [2, 7, 9, 11–13, 16, 20, 26, 40, 41, 43] and the references therein).

Towards this end, let (*M*, *g*) be a complete Riemannian manifold of dimension $n \ge 2$, $d\mu =e^{-f} dv_g$ be a weighted measure associated with the metric *g* and potential *f*, and let $dv_g$ be the usual Riemannain volume measure on *M*. The triple $(M, g, d\mu )$ is referred to as a smooth metric measure space. Our prime interest in this paper is the nonlinear fast diffusion equation (FDE for brevity) associated with the triple $(M,g,d\mu )$ that can be written in the explicit form:

1.1 $$\begin{aligned} \square _p u(x,t) = \partial _t u (x,t) - \Delta _f u^p (x,t) = {\mathscr {N}}(t,x,u(x,t)), \qquad 0<p<1. \end{aligned}$$

The context in which we consider this nonlinear FDE is one where the metric tensor *g* and potential *f* are generally allowed to be time dependent. Such problems have moved to the forefront of research in geometric analysis since the work of G. Perelman [29] and the study of forward and backward heat equations on manifolds evolving under (super) Ricci-Hamilton or (super) Ricci-Perelman flows. Naturally in this setting the weighted measure, the various curvature tensors and the familiar (metric dependent) differential operators are all time dependent which makes the analysis more complicated and more challenging. Before discussing this aspect of the problem further let us take a moment to describe the different components and ingredients in equation (1.1).

The operator $\Delta _f$ in (1.1) is the *f*-Laplacian (or weighted Laplacian) acting on functions $v \in \mathscr {C}^2(M)$ by the prescription

1.2 $$\begin{aligned} \Delta _f v = \textrm{div}_f \nabla v = e^f \textrm{div}(e^{-f} \nabla v) = \Delta v - \langle \nabla f, \nabla v\rangle . \end{aligned}$$

It is a symmetric diffusion operator with respect to the invariant weighted measure $d\mu$ and it arises in a variety of contexts ranging from probability theory and geometry to quantum field theory and statistical mechanics [7, 41]. The *f*-Laplacian $\Delta _f$ is a natural generalisation of the Laplace-Beltrami operator to the SMMS context and it coincides with the latter precisely when the potential *f* is spatially constant.

Referring to (1.1) again, ${{\mathscr {N}}}={{\mathscr {N}}}(t,x,u)$ is a sufficiently smooth *nonlinearity* or forcing term depending on the space-time variables (*x*, *t*) and the dependent variable (solution) $u>0$. The form and structural features of this nonlinearity will evidently have analytical and physical significances and one of our aims is to understand the way it appears in the estimates and the manner in which it influences the solutions. To the best of our knowledge our results here are the first to consider the role of nonlinearity in the FDE and its role in the estimates and maximum exponent range, even for the static case.^1^

As for the geometry and curvature properties of the triple $(M,g,d\mu )$ we have a hierarchy of second order symmetric tensor fields on *M* defined by,

1.3 $$\begin{aligned} {\mathscr {R}ic}^m_f(g):= {{\mathscr {R}}ic}(g) + \nabla \nabla f - \frac{\nabla f \otimes \nabla f}{m-n}, \qquad m \ge n \ge 2, \end{aligned}$$

called the Bakry-Èmery *m*-Ricci curvature tensors. Here ${{\mathscr {R}}ic}(g)$ is the usual Riemannain Ricci curvature tensor of *g*, $\nabla \nabla f=\textrm{Hess}(f)$ denotes the Hessian of *f*, and $m \ge n$ a dimension type constant which is **not necessarily an integer** (see [7]). For the sake of clarification, in relation to (1.3), when $m=n$, by convention *f* is only allowed to be a constant, thus giving ${{\mathscr {R}}ic}^m_f(g)={{\mathscr {R}}ic}(g)$. We also allow for $m = \infty$ in which case by formally passing to the limit in (1.3) we set,

1.4 $$\begin{aligned} {{\mathscr {R}}ic}_f(g) = {{\mathscr {R}}ic}(g) + \nabla \nabla f. \end{aligned}$$

The identity relating the *f*-Laplacian $\Delta _f$ to the Bakry-Émery Ricci curvature tensor ${{\mathscr {R}}ic}_f(g)$ is the weighted Bochner-Weitzenböck formula saying that for *v* of class ${{\mathscr {C}}}^3(M)$,

1.5 $$\begin{aligned} \frac{1}{2} \Delta _f |\nabla v|^2 - \langle \nabla v, \nabla \Delta _f v \rangle = |\nabla \nabla v|^2 + {{\mathscr {R}}ic}_f (\nabla v, \nabla v). \end{aligned}$$

As is readily seen (1.5) is not immediately applicable to the Bakry-Émery *m*-Ricci curvature tensor (1.3). However, by adding and subtracting the symmetric second order rank-one tensor $[\nabla f \otimes \nabla f]/(m-n)$ to (1.5) and an application of the Cauchy-Schwarz inequality one obtains the *inequality*

1.6 $$\begin{aligned} \frac{1}{2} \Delta _f |\nabla v|^2 - \langle \nabla v, \nabla \Delta _f v \rangle&\ge \frac{1}{n} (\Delta v)^2 + {{\mathscr {R}}ic}_f (\nabla v, \nabla v) \nonumber \\&\ge \frac{1}{m} (\Delta _f v)^2 + {{\mathscr {R}}ic}^m_f (\nabla v, \nabla v). \end{aligned}$$

As an easy inspection reveals, a lower bound on ${\mathscr {R}ic}_f^m(g)$, in the sense of symmetric tensors, is a stronger condition than the same lower bound on ${{\mathscr {R}}ic}_f(g)$, in that, we have the one way implication

1.7 $$\begin{aligned} {{\mathscr {R}}ic}_f^m(g) = {{\mathscr {R}}ic}_f(g) - \frac{\nabla f \otimes \nabla f}{m-n} \ge {{\textsf{k}}} g \implies {{\mathscr {R}}ic}_f(g) \ge {{\textsf{k}}} g, \end{aligned}$$

but not the other way around (here ${{\textsf{k}}} \in {\mathbb {R}}$, $m \ge n$ are finite constants). This observation is an important one throughout the analysis below especially from the point of view of comparison with similar results for linear heat-type equations. Also it is the curvature tensor ${{\mathscr {R}}ic}_f^m(g)$ that plays the main role in the estimates and results here.

In this paper we present ourselves with the task of developing gradient estimates of elliptic type also called Hamilton-Souplet-Zhang type, for positive solutions to (1.1). These will be established using different sets of ideas and will cover different exponent ranges in the fast diffusion regime. The remarkable fact that the maximum exponent range in these estimates turns to be independent of the evolution of metric and potential as well as the curvature bounds is in sharp contrast to other classes of gradient estimates and one interesting outcome of our analysis.

Following the standard theory of degenerate and singular diffusion (*see* [10, 16, 39]), in the FDE and SMMS context here we introduce the second order linear (space-time dependent variable coefficient) evolution operator

1.8 $$\begin{aligned} {\mathscr {L}} = \partial _t - p u^{p-1}(x,t) \Delta _f, \end{aligned}$$

where $u=u(x,t)$ is a positive (solution) to (1.1) and $0<p<1$. This operator plays an important role throughout the paper, particularly, in that, by suitably transforming *u*, say to *v* (*see* below), it follows from *u* being a positive solution to (1.1) that *v* is in turn a positive solution to a related equation involving the linear (heat-type) operator ${\mathscr {L}}$. See Sects. 3 and 8 for more and the discussion surrounding (1.11) at the end of this this section. The transformation taking *u* to *v* is sometimes called the pressure transform.

In this paper we prove two sets of gradient estimates of Hamilton-Souplet-Zhang type for positive solutions to (1.1). The first estimate formulated in Theorem 2.1 covers the **super-critical range** $p_c< p <1$ where $p_c=p_c(m) = 1-2/m$. The second estimate formulated in Theorem 7.1 covers the range $p_0<p<1$ where $p_0=p_0(m)$ is the larger of 1/2 and $1-1/\sqrt{m-1}$, i.e., $p_0(m)=\max (1/2, 1-1/\sqrt{m-1})$. It is not difficult to see that this latter range (depending on $m \ge 2$) covers both the super-critical range and partly the **very fast diffusion** or **sub-critical range** $0<p<p_c$. In what follows we refer to the larger of the ranges $p_c(m)<p<1$ or $p_0(m)<p<1$ as the **maximum exponent range** (for that particular *m*). Note that *m* is not necessarily an integer.^2^

Indeed, looking more closely at these exponents and noting $1-1/\sqrt{m-1} \le 1-2/m$ (for $m \ge 2$) it is easy to describe the maximum exponent range for different values of *m* (along with the corresponding theorem) in the way below^3^:

- $$2 \le m \le 4$$: $$p_c \le p_0=1/2 \implies $$ $$p_c<p<1$$ (Theorem 2.1).
- $$4 \le m \le 5$$: $$1-1/\sqrt{m-1} \le 1/2 =p_0 \le p_c \implies $$ $$p_0<p<1$$ (Theorem 7.1).
- $$m \ge 5$$: $$1/2 \le 1-1/\sqrt{m-1} = p_0 \le p_c \implies $$ $$p_0<p<1$$ (Theorem 7.1).

A remarkable outcome of our analysis is that although the evolution of the metric *g* and potential *f* and the analytic properties of the nonlinearity ${{\mathscr {N}}}$ directly influence the estimate and bounds, however, neither the nonlinearity nor the evolution of the metric and potential, have any influence on the maximum exponent range in the estimates.

The evolution operator (1.8) and the corresponding transformation from *u* to *v* takes different forms in each of the two sets of estimates and corresponding theorems:

- (Sect. 2: Theorems 2.1 to 2.5, Sect. 6) For the range $0 \le p_c(m)<p<1$ we have $v= p/(1-p) u^{p-1}$ and subsequently
  1.9 $$\begin{aligned} {\mathscr {L}} = \partial _t-(1-p ) v \Delta _f. \end{aligned}$$
- (Sect. 7: Theorems 7.1 to 7.5, Sect. 11) For the range $1/2 \le p_0(m)<p<1$ we have $v=u^{p-1/2}$ and subsequently
  1.10 $$\begin{aligned} {\mathscr {L}} = \partial _t - pv^{2(p-1)/(2p-1)} \Delta _f. \end{aligned}$$

An interesting fact to highlight here is that in the nonlinear diffusion context studied in this paper, the metric and potential super flow inequality

1.11 $$\begin{aligned} \frac{1}{2} \partial _t g(x,t) + p u^{p-1}(x,t) {\mathscr {R}ic}_f^m(g)(x,t) \ge - {{\textsf{k}}} g(x,t) \end{aligned}$$

presents itself as the substitute for the super Ricci-Perelman flow inequality that links to linear heat-type equations where $p=1$ (see [33–35, 37]). As will become evident later, this formulation is closely connected with the evolution operator ${{\mathscr {L}}}$ defined in (1.8) and reduces to the usual Ricci-Perelman super flow when $p=1$. Again to make the distinction between the two sets of estimates considered here clearer, we point out that, the super flow (1.11) in each of the two cases above takes the form:

- For $0\le p_c(m)<p<1$ with transformation $v=p/(1-p) u^{p-1}$ we have
  1.12 $$\begin{aligned} \frac{1}{2} \dfrac{\partial g}{\partial t} (x,t) + (1-p) v(x,t) {{\mathscr {R}}ic}^m_f(g)(x,t) \ge - \textsf{k}g(x,t), \qquad {\mathsf k} \ge 0. \end{aligned}$$
- For $1/2 \le p_0(m)<p<1$ with transformation $v=u^{p-1/2}$ we have
  1.13 $$\begin{aligned} \frac{1}{2} \dfrac{\partial g}{\partial t} (x,t) + p v^{2(p-1)/(2p-1)}(x,t) {{\mathscr {R}}ic}^m_f(g)(x,t) \ge - \textsf{k}g(x,t), \qquad {\textsf{k}} \ge 0. \end{aligned}$$

As a further comment we also note that in the main results and estimates in Theorem 2.1 and Theorem 7.1 we shall make use of the lower bounds

1.14 $$\begin{aligned} \partial _t g (x,t) \ge - 2h g(x,t), \qquad {{\mathscr {R}}ic}^m_f(g)(x,t) \ge - k g(x,t), \end{aligned}$$

for suitable constants *h*, $k \ge 0$ in a fixed compact space-time cylinder $Q_{R,T}$ with upper base centered at the reference point where the estimate is sought. These bounds on the one hand allow for the use of weighted (Laplace) comparison theorems and on the other provide control on the time derivative of geodesic distances which are two important ingredients in localisation and the proof of gradient estimates. The constants *h*, $k \ge 0$ will appear along with other bounds in the geometry-dependent terms in the ultimate formulation of the local estimates. In the static case, where the metrics and potentials do not depend on time (i.e., $\partial _t g \equiv 0$ and $\partial _t f \equiv 0$) we can take $h=0$ and our results are immediately seen to cover this special case with the usual assumption of Ricci curvature lower bound (space only) of the type ${{\mathscr {R}}ic}_f^m(g) \ge - {{\textsf{k}}} g$.

**Plan of the paper.** The main estimates are presented in Theorem 2.1 in Sect. 2 and Theorem 7.1 in Sect. 7 where both estimates are formulated in their local forms. These estimates cover different exponent ranges and their proofs rely on different set of techniques. The global forms of these estimates follow by imposing appropriate global bounds on the solution, metric, potential as well as the Bakry-Émery *m*-Ricci curvature tensor and is formulated in Theorem 2.3 and Theorem 7.3 respectively. The static case (time independent metrics *g* and potentials *f*) which is an important case is treated as a by-product of the above estimates and is presented in Theorem 2.4 and Theorem 7.4 respectively. As a nice and useful application of these we are able to establish parabolic Liouville results which lead to a characterisation of ancient solutions to (1.1). These appear in turn in Theorem 2.5 and Theorem 7.5. As such Sects. 2 and 7 contain the main results of the paper whilst the remaining sections are devoted to developing the necessary apparatus and tools for establishing the results and proofs. Sections 6 and 11 present another new set of global bounds and estimates by direct consideration of the super flow (1.11) [in the forms (1.12) and (1.13)] and suitable application of maximum principle. Here *M* is taken to be closed.

**Notation.** We write $z=z_+ + z_-$ with $z_+=\max (z, 0)$ and $z_-=\min (z, 0)$. Fixing a reference point $x_0 \in M$ we denote by $d=d(x,x_0, t)$ the Riemannian distance between *x* and $x_0$ on *M* with respect to $g=g(t)$. We write $\varrho =\varrho (x,x_0,t)$ for the geodesic radial variable measuring distance between *x* and origin $x_0$ at time *t*. For a fixed space-time reference point $(x_0,t_0)$ and $R>0$, $T>0$ we define the space-time cylinder

1.15 $$\begin{aligned} Q_{R,T} (x_0,t_0) \equiv \{ (x, t) | d(x, x_0, t) \le R, t_0-T \le t \le t_0 \} \subset M \times [t_0-T, t_0]. \end{aligned}$$

When the metric *g* is time independent, we denote by $\mathscr {B}_\varrho (x_0) \subset M$ the geodesic ball of radius $\varrho >0$ centered at $x_0$. It is evident that in this case we have

1.16 $$\begin{aligned} Q_{R,T} (x_0,t_0) = \mathscr {B}_R(x_0) \times [t_0-T, t_0] \subset M \times [t_0-T, t_0]. \end{aligned}$$

When the choice of the reference point is clear from the context we often abbreviate and write *d*(*x*, *t*), $\varrho (x,t)$ or ${\mathscr {B}}_\varrho$, $Q_{R,T}$ respectively. For a function of multiple variables, we denote its partial derivatives with subscripts accordingly. For instance for $\Gamma =\Gamma (x,u)$ we denote its partial derivatives with respect to $x=(x_1, \dots , x_n)$ or *u* by $\Gamma _x$ or $\Gamma _u$.

## A Hamilton-Souplet-Zhang estimate in the range $p_c(m)< p < 1$

In this section we present the first set of gradient estimates for positive solutions to (1.1). These cover the range $p_c< p<1$. Here $p_c=p_c(m)\ge 0$ is the critical exponent defined by $p_c=1-2/m$ where $m \ge 2$ (and is not necessarily an integer). Denoting by $\beta _1$, $\beta _2$ the roots of the quadratic polynomial $\beta ^2 - (2-p)/(1-p)\beta +m/2$, we pick $\beta$ such that $0< \beta _1<\beta \le 1<\beta _2$, i.e., $\beta \in (\beta _1, \beta _2)$ and $\beta \le 1$. Additionally throughout we use the notation

2.1 $$\begin{aligned} \Sigma (t,x,v)= p [(1-p)v/p]^{\frac{2-p}{1-p}} {\mathscr {N}} \left( t,x,[(1-p)v/p]^{\frac{1}{p-1}}\right) , \end{aligned}$$

for the scaled nonlinearity resulting from the change from *u* to *v* (see below).

### Theorem 2.1

Let $(M, g, d\mu )$ be a complete smooth metric measure space with $d\mu =e^{-f} dv_g$. Assume the metric and potential are time dependent, of class $\mathscr {C}^2$ and that for suitable constants $k, h \ge 0$ and $m \ge n$ satisfy ${{\mathscr {R}}ic}_f^m (g) \ge -(m-1)k g$, $\partial _t g \ge -2hg$ in the compact space-time cylinder $Q_{R,T}$ with $R, T >0$. Let *u* be a positive solution to (1.1) with $p_c< p < 1$ and let $v =p/(1-p) u^{p-1}$ and $M= \sup _{Q_{R,T}} v$. Then there exists $C=C(p,\beta ,m)>0$ such that for every (*x*, *t*) in $Q_{R/2,T}$ with $t>t_0-T$ we have

2.2 $$\begin{aligned} \frac{|\nabla v|}{v^{\beta /2}} (x,t) \le C \left\{ \begin{array}{ll} \sqrt{h} M^{(1-\beta )/2} + \left[ \dfrac{k^{1/4}}{\sqrt{R}} + \dfrac{1}{R} + \sqrt{k} \right] M^{1-\beta /2} \\ \\ + \dfrac{M^{(1-\beta )/2}}{\sqrt{t-t_0+T}} + \sup _{Q_{R, T}} \left\{ \left[ \dfrac{|\Sigma _x(t,x,v)|}{v^{(3\beta -2)/2}}\right] ^{1/3}\right\} \\ \\ + \sup _{Q_{R, T}}\left\{ v^{(1-\beta )/2}\left[ \dfrac{ \beta \Sigma (t,x,v)}{v}-2 \Sigma _v(t,x,v) \right] _+^{1/2}\right\} \end{array} \right\} . \end{aligned}$$

### Remark 2.2

Since *u* and *v* are here related to one-another via $u =[(1-p)v/p]^{1/(p-1)}$ we can write (2.1) as $\Sigma (t,x,v) = p u^{p-2} {{\mathscr {N}}}(t,x,u)$.

Subject to global bounds we have the following global estimate (in space) that results from passing to the limit $R \rightarrow \infty$ in (2.2).

### Theorem 2.3

Let $(M, g, d\mu )$ be a complete smooth metric measure space with $d\mu =e^{-f} dv_g$. Assume the metric and potential are time dependent, of class $\mathscr {C}^2$ and that for suitable constants $k, h \ge 0$ and $m \ge n$ satisfy ${{\mathscr {R}}ic}_f^m (g) \ge -(m-1)k g$, $\partial _t g \ge -2hg$ on $M \times [t_0-T, t_0]$. Let *u* be a positive solution to (1.1) with $p_c< p < 1$ and let $v =p/(1-p) u^{p-1}$ and $M= \sup v$. Then there exists $C=C(p,\beta ,m)>0$ such that for every $x \in M$ and $t_0-T<t\le t_0$ we have

2.3 $$\begin{aligned} \frac{|\nabla v|}{v^{\beta /2}} (x,t) \le C \left\{ \begin{array}{ll} \sqrt{k} M^{1-\beta /2} + \left[ \sqrt{h} + \dfrac{1}{\sqrt{t-t_0+T}} \right] M^{(1-\beta )/2} \\ \\ + \sup _{M \times [t_0-T, t_0]} \left\{ \left[ \dfrac{|\Sigma _x(t,x,v)|}{v^{(3\beta -2)/2}}\right] ^{1/3}\right\} \\ \\ + \sup _{M \times [t_0-T, t_0]} \left\{ v^{(1-\beta )/2} \left[ \dfrac{ \beta \Sigma (t,x,v)}{v}-2 \Sigma _v(t,x,v) \right] _+^{1/2}\right\} \end{array} \right\} . \end{aligned}$$

The static case ($\partial _t g \equiv 0$ and $\partial _t f \equiv 0$) is of enough significance to be formulated as a separate corollary. Here we describe the local version. The global version follows by passing to the limit $R\rightarrow \infty$.

### Theorem 2.4

Let $(M, g, d\mu )$ be a complete smooth metric measure space with $d\mu =e^{-f} dv_g$ and assume ${{\mathscr {R}}ic}_f^m (g) \ge -(m-1)k g$ in ${{\mathscr {B}}}_R$ for some $k \ge 0$, $m \ge n$ and $R>0$. Let *u* be a positive solution to (1.1) with $p_c< p < 1$ and let $v =p/(1-p) u^{p-1}$ and $M= \sup _{Q_{R,T}} v$. Then there exists $C=C(p,\beta ,m)>0$ such that for every (*x*, *t*) in $Q_{R/2,T}$ with $t>t_0-T$ we have

2.4 $$\begin{aligned} \frac{|\nabla v|}{v^{\beta /2}} (x,t) \le C \left\{ \begin{array}{ll} \left[ \dfrac{k^{1/4}}{\sqrt{R}} + \dfrac{1}{R} + \sqrt{k} \right] M^{1-\beta /2} \\ \\ + \dfrac{M^{(1-\beta )/2}}{\sqrt{t-t_0+T}} + \sup _{Q_{R, T}} \left\{ \left[ \dfrac{|\Sigma _x(t,x,v)|}{v^{(3\beta -2)/2}}\right] ^{1/3}\right\} \\ \\ + \sup _{Q_{R, T}}\left\{ v^{(1-\beta )/2}\left[ \dfrac{ \beta \Sigma (t,x,v)}{v}-2 \Sigma _v(t,x,v) \right] _+^{1/2}\right\} \end{array} \right\} . \end{aligned}$$

We end this section with an application of the estimates above to parabolic Liouville-type theorems. Here by an ancient solution $u=u(x,t)$ to (1.1) we mean a solution defined on *M* for all negative times, that is, for all (*x*, *t*) with $x \in M$ and $-\infty< t <0$.

### Theorem 2.5

Let $(M, g, d\mu )$ be a complete smooth metric measure space with $d\mu =e^{-f}dv_g$ and ${{\mathscr {R}}ic}^m_f(g) \ge 0$. Assume $[(2-p)-\beta (1-p)/2] {{\mathscr {N}}}(u)/u - {{\mathscr {N}}}_u(u) \ge 0$ for some $\beta \in (\beta _1, \beta _2)$ and all $u>0$ where $p_c< p<1$. Then any positive ancient solution to the nonlinear fast diffusion equation

2.5 $$\begin{aligned} \square _p u = \partial _t u - \Delta _f u^p = {{\mathscr {N}}}(u(x,t)), \end{aligned}$$

satisfying the growth at infinity $1/u(x,t) = o( [\varrho (x) + \sqrt{|t|}]^{2/[(1-p)(2-\beta )]})$ must be spatially constant. If, in addition, ${{\mathscr {N}}}(u) \ge a$ for some $a>0$ and all $u>0$ then (2.5) admits no such ancient solutions.

## Evolution inequalities $\textbf{I}$: ${\mathscr {L}} = \partial _t -(1-p) v \Delta _f$ acting on $w = |\nabla v|^2/v^{\beta }$

In this section we derive evolution identities and inequalities for $w = |\nabla v|^2/v^{\beta }$ where $v =p/(1-p) u^{p-1}$. The evolution operator is ${\mathscr {L}} = \partial _t -(1-p) v \Delta _f$ and $\beta \in {\mathbb {R}}$ is fixed. Note that in the next few lemmas we assume $0<p<1$ but later in the proof of the estimates in Sect. 4 we further restrict this range to $1-2/m=p_c<p<1$.

### Lemma 3.1

Let *u* be a positive solution to (1.1) with $0<p<1$, set $v =p/(1-p) u^{p-1}$ and let $\Sigma =\Sigma (t,x,v)$ be as in (2.1). Then *v* satisfies the evolution equation

3.1 $$\begin{aligned} {\mathscr {L}} [v] = [\partial _t -(1-p) v \Delta _f]v = - |\nabla v|^2 - \Sigma (t,x,v). \end{aligned}$$

### Proof

As here *u* and *v* are related via $u = [(1-p)v/p]^{1/(p-1)}$ basic calculations give

3.2 $$\begin{aligned} \partial _t u&=\partial _t [(1-p)v/p]^{1/(p-1)} = -(1/p) [(1-p)v/p]^{\frac{2-p}{p-1}} \partial _t v, \nonumber \\ \Delta _f u^p&= -(1/p) [(1-p)v/p]^{\frac{2-p}{p-1}}[(1-p) v \Delta v - |\nabla v|^2] + [(1-p)v/p]^{\frac{1}{p-1}}\langle \nabla f , \nabla v \rangle . \end{aligned}$$

Now substituting these ingredients into (1.1) results in

3.3 $$\begin{aligned} \square _p u = \partial _t u - \Delta _f u^p&= -(1/p) [(1-p)v/p]^{\frac{2-p}{p-1}} (\partial _t v - (1-p)v \Delta _f v + |\nabla v|^2) \nonumber \\&= {\mathscr {N}} \left( t,x,[(1-p)v/p]^{\frac{1}{p-1}}\right) \end{aligned}$$

which by recalling (2.1) and rearranging terms gives at once the desired conclusion. $\square$

### Lemma 3.2

Let $(M, g, d\mu )$ be a complete smooth metric measure space with $d\mu =e^{-f} dv_g$. Suppose that the metric and potential are time dependent and of class $\mathscr {C}^2$. Let *u* be a positive solution to (1.1) with $0<p<1$ and $w = |\nabla v|^2/v^{\beta }$ where $v =p/(1-p) u^{p-1}$ and $\beta \in {\mathbb {R}}$ is a constant. Then *w* satisfies the evolution equation

3.4 $$\begin{aligned} {\mathscr {L}} [w] =&-[\partial _t g+2(1-p)v{{\mathscr {R}}ic}_f^m(g)]\frac{( \nabla v, \nabla v)}{v^\beta }\nonumber \\&-\frac{2(1-p)}{v^{\beta }} \left[ v|\nabla \nabla v|^2 +\frac{v \langle \nabla f , \nabla v \rangle ^2}{(m-n)}-|\nabla v|^2 \Delta _f v\right] \nonumber \\&-2[1-\beta (1-p)] \frac{\langle \nabla v, \nabla |\nabla v|^2 \rangle }{v^\beta } +\beta \frac{ |\nabla v|^2 \Sigma (t,x,v)}{v^{\beta +1}}\nonumber \\&-\beta [(1-p)(\beta +1)-1]\frac{|\nabla v|^4}{v^{\beta +1}} -2 \frac{\langle \nabla v, \nabla \Sigma (t,x,v)\rangle }{v^{\beta }}. \end{aligned}$$

### Proof

Starting with $w = |\nabla v|^2/v^{\beta }$ and using equation (3.1) for *v* we can write

3.5 $$\begin{aligned} \partial _t w =&~ \partial _t \left[ \frac{|\nabla v|^2}{v^\beta }\right] = 2\frac{\langle \nabla v, \nabla \partial _t v \rangle }{v^\beta } -\frac{[\partial _t g] ( \nabla v, \nabla v)}{v^\beta } - \beta \frac{v^{\beta -1}|\nabla v|^2 \partial _t v}{v^{2\beta }} \nonumber \\ =&~ \frac{2\langle \nabla v ,\nabla [(1-p) v \Delta _f v -|\nabla v|^2 - \Sigma (t,x,v)]\rangle }{v^\beta } -\frac{[\partial _t g] ( \nabla v, \nabla v)}{v^\beta } \nonumber \\&-\frac{\beta |\nabla v|^2 [(1-p ) v \Delta _f v -|\nabla v|^2 - \Sigma (t,x,v)]}{v^{\beta +1}}\nonumber \\ =&~ 2(1-p)\frac{ |\nabla v|^2 \Delta _f v}{v^\beta } +2(1-p) \frac{\langle \nabla v, \nabla \Delta _f v \rangle }{v^{\beta -1}} - 2\frac{\langle \nabla v , \nabla |\nabla v|^2 \rangle }{v^\beta } + \beta \frac{ |\nabla v|^4}{v^{\beta +1}}\nonumber \\&- 2 \frac{\langle \nabla v, \nabla \Sigma (t,x,v)\rangle }{v^\beta } -\frac{[\partial _t g] ( \nabla v, \nabla v)}{v^\beta } - \beta (1-p)\frac{|\nabla v|^2 \Delta _f v}{v^\beta } + \beta \frac{|\nabla v|^2 \Sigma (t,x,v)}{v^{\beta +1}}. \end{aligned}$$

Next using $\nabla w = \nabla |\nabla v|^2/v^\beta - \beta |\nabla v|^2 \nabla v/v^{\beta +1}$ and $\Delta _f v = \Delta v - \langle \nabla f, \nabla v\rangle$ we have

3.6 $$\begin{aligned} \Delta _f w = \frac{\Delta _f |\nabla v|^2}{v^\beta } - 2 \beta \frac{\langle \nabla v, \nabla |\nabla v|^2 \rangle }{v^{\beta +1}} - \beta \frac{|\nabla v|^2 \Delta _f v}{v^{\beta +1}} +\beta (\beta +1) \frac{ |\nabla v|^4}{v^{\beta +2}}. \end{aligned}$$

Putting (3.5)-(3.6) together, simplifying and rearranging terms then gives

3.7 $$\begin{aligned} [\partial _t-(1-p) v \Delta _f] w =&-(1-p) \frac{\Delta _f |\nabla v|^2}{v^{\beta -1}} -2[1- \beta (1-p)] \frac{\langle \nabla v, \nabla |\nabla v|^2 \rangle }{v^\beta }\nonumber \\&- \beta [(1-p)(\beta +1)-1]\frac{|\nabla v|^4}{v^{\beta +1}} +2(1-p) \frac{|\nabla v|^2 \Delta _f v}{v^{\beta }}\nonumber \\&+2(1-p) \frac{\langle \nabla v, \nabla \Delta _f v \rangle }{v^{\beta -1}} -\frac{[\partial _t g] ( \nabla v, \nabla v)}{v^\beta } \nonumber \\&-2 \frac{\langle \nabla v, \nabla \Sigma (t,x,v)\rangle }{v^{\beta }} + \beta \frac{ |\nabla v|^2 \Sigma (t,x,v)}{v^{\beta +1}}. \end{aligned}$$

An application of the weighted Bochner-Weitzenböck formula (1.5) to the sum of the first and fifth terms on the right-hand side in (3.7) leads to

3.8 $$\begin{aligned} (3.7) =&-\frac{2(1-p)}{v^{\beta -1}}\left[ |\nabla \nabla v|^2 +{{\mathscr {R}}ic}_f^m (\nabla v, \nabla v)+\frac{\langle \nabla f , \nabla v \rangle ^2}{(m-n)}\right] \nonumber \\&-2[1- \beta (1-p)] \frac{\langle \nabla v, \nabla |\nabla v|^2 \rangle }{v^\beta } -\beta [(1-p)(\beta +1)- 1]\frac{|\nabla v|^4}{v^{\beta +1}}\nonumber \\&-\frac{[\partial _t g] ( \nabla v, \nabla v)}{v^\beta }+2(1-p) \frac{|\nabla v|^2 \Delta _f v}{v^{\beta }} -2 \frac{\langle \nabla v, \nabla \Sigma (t,x,v) \rangle }{v^{\beta }} +\beta \frac{ |\nabla v|^2 \Sigma (t,x,v)}{v^{\beta +1}}. \end{aligned}$$

A rearrangement of terms now gives the desired identity. $\square$

### Lemma 3.3

Under the assumptions of Lemma 3.2, *w* satisfies the evolution inequality

3.9 $$\begin{aligned} {\mathscr {L}} [w] \le&-[\partial _t g + 2(1-p) v {{\mathscr {R}}ic}_f^m (g)] \frac{(\nabla v, \nabla v)}{v^\beta } \nonumber \\&- 2[1- \beta (1-p)] \langle \nabla v, \nabla w \rangle +(1-p) \left[ \beta ^2 -\frac{2-p}{1-p}\beta +\frac{m}{2} \right] v^{\beta -1} w^2 \nonumber \\&- 2 \frac{\langle \nabla v, \Sigma _x(t,x,v) \rangle }{v^\beta } +\left[ \frac{ \beta \Sigma (t,x,v)}{v}-2 \Sigma _v(t,x,v) \right] w. \end{aligned}$$

### Proof

Starting from (3.4), making note of $\langle \nabla v, \nabla |\nabla v|^2 \rangle /v^\beta = \langle \nabla v, \nabla w \rangle +\beta |\nabla v|^4/v^{\beta +1}$ and $\langle \nabla v, \nabla \Sigma (t,x,v) \rangle =\langle \nabla v, \Sigma _x(t,x,v) \rangle + \Sigma _v(t,x,v) |\nabla v|^2$ we have after substitution

3.10 $$\begin{aligned} {[}\partial _t - (1-p) v\Delta _f] w =&-[\partial _t g+2(1-p)v{{\mathscr {R}}ic}_f^m(g)]\frac{( \nabla v, \nabla v)}{v^\beta }\nonumber \\&-\frac{2(1-p)}{v^{\beta }} \left[ v|\nabla \nabla v|^2 +\frac{v \langle \nabla f , \nabla v \rangle ^2}{(m-n)}-|\nabla v|^2 \Delta _f v\right] \nonumber \\&-2[1-\beta (1-p)] \langle \nabla v, \nabla w \rangle +\left[ \beta \frac{ \Sigma (t,x,v)}{v}-2 \Sigma _v(t,x,v)\right] \frac{|\nabla v|^2}{v^{\beta }}\nonumber \\&+[(1-p)\beta ^2-(2-p)\beta ]\frac{|\nabla v|^4}{v^{\beta +1}} -2\frac{\langle \nabla v, \Sigma _x(t,x,v) \rangle }{v^\beta }. \end{aligned}$$

Next, using the inequality $|\nabla \nabla v|^2 + \langle \nabla f, \nabla v \rangle ^2/(m-n) \ge (\Delta _f v)^2/m$, it is seen that

3.11 $$\begin{aligned}&-\frac{2(1-p)}{v^{\beta -1}} \left[ |\nabla \nabla v|^2 + \frac{\langle \nabla f , \nabla v \rangle ^2}{m-n} - \frac{|\nabla v|^2 \Delta _f v}{v}\right] \le -\frac{2(1-p)}{v^{\beta -1}} \left[ \frac{(\Delta _f v)^2}{m} - \frac{|\nabla v|^2 \Delta _f v}{v}\right] \nonumber \\&\qquad \le - \frac{2(1-p)}{v^{\beta -1}} \left[ \left( \frac{\Delta _f v}{\sqrt{m}} - \frac{\sqrt{m}}{2} \frac{|\nabla v|^2}{v} \right) ^2 -\frac{m}{4} \frac{|\nabla v|^4}{v^2} \right] \le \frac{m(1-p)}{2} \frac{|\nabla v|^4}{v^{\beta +1}}. \end{aligned}$$

Therefore substituting the above in (3.10), making the substitution $w = |\nabla v|^2/v^{\beta }$ and rearranging terms gives the desired conclusion. $\square$

### Lemma 3.4

Under the assumptions of Lemma 3.3, if for some ${{\textsf{k}}} \ge 0$ and $m \ge n$ the metric and potential satisfy the super flow

3.12 $$\begin{aligned} \frac{1}{2} \partial _t g + (1-p) v {{\mathscr {R}}ic}_f^m (g) \ge - {{\textsf{k}}} g, \end{aligned}$$

then *w* satisfies the evolution inequality

3.13 $$\begin{aligned} {\mathscr {L}} [w] \le&~2 {{\textsf{k}}} w - 2[1- \beta (1-p)] \langle \nabla v, \nabla w \rangle - 2 \frac{\langle \nabla v, \Sigma _x(t,x,v) \rangle }{v^\beta } \nonumber \\&+(1-p) \left[ \beta ^2 -\frac{2-p}{1-p}\beta +\frac{m}{2} \right] v^{\beta -1} w^2 +\left[ \frac{ \beta \Sigma (t,x,v)}{v}-2 \Sigma _v(t,x,v) \right] w. \end{aligned}$$

### Proof

This follows immediately from Lemma 3.3 by substituting (3.12) into (3.9) and using $w = |\nabla v|^2/v^{\beta }$. $\square$

### Remark 3.5

Note that under the assumptions ${{\mathscr {R}}ic}_f^m(g) \ge -(m-1) kg$ and $\partial _t g \ge - 2\,h g$ (as in Theorem 2.1) we can replace ${{\textsf{k}}}$ in (3.13) with $(1-p)(m-1)kv+h$ or subject to $0<v \le M$ (again as in Theorem 2.1) with $(1-p)(m-1)kM+h$.

## Localisation in space-time cylinders $Q_{R,T}$ and proof of Theorem 2.1

As the gradient estimate in Theorem 2.1 is a local estimate, its proof is naturally based on localisation and requires the construction of suitable cut-off functions. To this end, let us fix a base point $x_0 \in M$, a reference time $t_0 \in {\mathbb {R}}$, and $R, T>0$ and then $\tau \in (t_0-T, t_0]$. The following standard lemma grants the existence of a smooth function $\bar{\eta }=\bar{\eta }(\varrho , t)$ of two real variables $\varrho \ge 0$ and $t_0-T \le t \le t_0$ respectively satisfying a set of useful bounds and properties for carrying out the *cylindrical* localisation procedure. The desired cut-off function $\eta$ will then be constructed from ${\bar{\eta }}$ via (4.2).

### Lemma 4.1

Fix $t_0 \in {\mathbb {R}}$ and let $R, T>0$. Given $\tau \in (t_0-T, t_0]$ there exists a smooth function $\bar{\eta }:[0,\infty ) \times [t_0-T, t_0] \rightarrow \mathbb {R}$ such that the following properties hold:

(i) $$\textrm{supp} \, \bar{\eta }(\varrho ,t) \subset [0,R] \times [t_0-T, t_0]$$ and $$0 \le \bar{\eta }(\varrho ,t) \le 1$$ in $$[0,R] \times [t_0-T, t_0]$$,
(ii) $\bar{\eta }=1$ in $[0,R/2] \times [\tau , t_0]$ and $\partial \bar{\eta }/\partial \varrho =0$ in $[0,R/2] \times [t_0-T, t_0]$, respectively,
(iii) there exists $c>0$ such that
  4.1 $$\begin{aligned} \frac{|\partial _t \bar{\eta }|}{\sqrt{{\bar{\eta }}}} \le \frac{c}{\tau -t_0+T}, \end{aligned}$$
  in $[0,\infty )\times [t_0-T,t_0]$ and $\bar{\eta }(\varrho ,t_0-T)=0$ for all $\varrho \in [0,\infty )$.
(iV) $-c_a \bar{\eta }^a/R \le \partial _\varrho \bar{\eta } \le 0$ and $|\partial _{\varrho \varrho } \bar{\eta }| \le c_a \bar{\eta }^a / R^2$ hold on $[0, \infty )\times [t_0-T, t_0]$ for every $0<a<1$ and some $c_a>0$.

Having the above lemma in place we now move on to introducing a smooth cylindrical cut-off function $\eta =\eta (x,t)$ by setting, for $R>0$, $T>0$ and $t_0-T <\tau \le t_0$,

4.2 $$\begin{aligned} \eta (x,t) = \bar{\eta }(\varrho (x,t), t), \end{aligned}$$

for $(x, t) \in M \times [t_0-T, t_0]$. Referring to (4.2) a straightforward calculation gives

- $$\partial _t \eta = \partial _\varrho {\bar{\eta }} \partial _t \varrho + \partial _t {\bar{\eta }}$$,
- $$\nabla \eta = \partial _\varrho {\bar{\eta }} \nabla \varrho $$, and,
- $$\Delta _f \eta = \partial _{\varrho \varrho } {\bar{\eta }} |\nabla \varrho |^2 + \partial _\varrho {\bar{\eta }} \Delta _f \varrho $$.

As a result we have from the above:

4.3 $$\begin{aligned} {\mathscr {L}} [\eta ]&= [\partial _t - (1-p)v \Delta _f] \eta \nonumber \\&= \partial _\varrho {\bar{\eta }} \partial _t \varrho + \partial _t {\bar{\eta }} - (1-p) v [\partial _{\varrho \varrho } {\bar{\eta }} |\nabla \varrho |^2 + \partial _\varrho {\bar{\eta }} \Delta _f \varrho ] \nonumber \\&= \partial _\varrho {\bar{\eta }} {\mathscr {L}} [\varrho ] + [\partial _t - (1-p) v |\nabla \varrho |^2 \partial _{\varrho \varrho }] {\bar{\eta }}. \end{aligned}$$

With this introduction we now proceed onto completing the proof of Theorem 2.1. To this end by considering the localised function $\eta w$ we have

4.4 $$\begin{aligned} {\mathscr {L}} [\eta w] = w{\mathscr {L}} [\eta ] - 2(1-p)v [\langle \nabla \eta ,\nabla (\eta w) \rangle - |\nabla \eta |^2 w]/\eta +\eta {\mathscr {L}} [w]. \end{aligned}$$

Using (3.13) in Lemma 3.4 and $\eta \langle \nabla v, \nabla w \rangle = \langle \nabla v, \nabla (\eta w) \rangle - w \langle \nabla v, \nabla \eta \rangle$ we can write

4.5 $$\begin{aligned} {\mathscr {L}} [\eta w] \le&~ w {\mathscr {L}} [\eta ] -2(1-p) v \left\langle \frac{\nabla \eta }{\eta } , \nabla (\eta w) \right\rangle + 2(1-p)v \frac{|\nabla \eta |^2}{\eta } w \nonumber \\&+ 2[(1-p) (m-1)k v + h] \eta w -2 [1-\beta (1-p)] \langle \nabla v , \nabla (\eta w) \rangle \nonumber \\&+2 [1-\beta (1-p)] w\langle \nabla v , \nabla \eta \rangle + (1-p) \left[ \beta ^2 -\frac{2-p}{1-p} \beta +\frac{m}{2} \right] v^{\beta -1} \eta w^2\nonumber \\&- 2\eta \frac{\langle \nabla v, \Sigma _x(t,x,v) \rangle }{v^\beta } + \left[ \frac{ \beta \Sigma (t,x,v)}{v} -2 \Sigma _v(t,x,v)\right] \eta w. \end{aligned}$$

Assume now that the localised function $\eta w$ achieves its maximum over the compact set $\{(x,t) | d(x,x_0, t) \le R, t_0-T \le t \le \tau \} \subset M \times [t_0-T, t_0]$ at the point $(x_1, t_1)$. We assume that $(\eta w)(x_1, t_1) >0$ as otherwise the conclusion of the theorem holds true with $w(x, \tau ) \le 0$ for all $d(x, x_0, \tau ) \le R/2$. Since $\eta \equiv 0$ when $t=t_0-T$ by part (*iii*) in Lemma 4.1, it therefore follows from this strict inequality that $t_1>t_0-T$. Hence at the maximum point $(x_1,t_1)$ we have the conditions

- $$\partial _t (\eta w) \ge 0$$,
- $$\nabla (\eta w) =0$$,
- $$\Delta _f(\eta w) \le 0$$,

and subsequently due to $v>0$ and $0<p<1$ we also have

4.6 $$\begin{aligned} {\mathscr {L}} [\eta w] = [\partial _t-(1-p)v \Delta _f] (\eta w) \ge 0. \end{aligned}$$

Now returning to (4.5), making use of the above and rearranging terms it is seen that at the maximum point $(x_1, t_1)$ we have

4.7 $$\begin{aligned} - (1-p) \left[ \beta ^2 -\frac{2-p}{1-p} \beta +\frac{m}{2} \right] v^{\beta -1} \eta w^2 \le&~ 2[(1-p)(m-1) k v + h] \eta w\nonumber \\&+ 2 [1-\beta (1-p)] w\langle \nabla v , \nabla \eta \rangle \nonumber \\&+ 2(1-p)v \frac{|\nabla \eta |^2}{\eta } w + w {\mathscr {L}} [\eta ]\nonumber \\&+\left[ \frac{ \beta \Sigma (t,x,v)}{v}-2 \Sigma _v(t,x,v) \right] \eta w\nonumber \\&- 2\eta \frac{\langle \nabla v, \Sigma _x(t,x,v) \rangle }{v^\beta }. \end{aligned}$$

We now need to choose $\beta$ such that firstly $\beta ^2 -(2-p)/(1-p) \beta +m/2<0$ and secondly that $\beta \le 1$ (the reason for this will become clearer shortly). As for the first condition we need $[(2-p)/(1-p)]^2>2m$ so that there are two roots $\beta _1< \beta _2$ to the quadratic polynomial in $\beta$ and as for the second condition we need $\beta _1<1$. These two conditions can be simultaneously satisfied *iff* $p \in (1-2/m,1)$. Picking $\beta \in (\beta _1, \beta _2)$ where the roots $\beta _1$, $\beta _2$ now satisfy $0<\beta _1<1<\beta _2$, we can set $-(1-p)[\beta ^2 -(2-p)/(1-p) \beta +m/2] =2/ \gamma$ with $\gamma > 0$.

Returning to (4.7) and multiplying through by $\gamma v^{1-\beta }$ we arrive at the following inequality at the maximum point $(x_1, t_1)$

4.8 $$\begin{aligned} 2 \eta w^2 \le&~ 2 \gamma [(1-p)(m-1) k v^{2-\beta } + h v^{1-\beta }]\eta w\nonumber \\&+ 2 \gamma [1-\beta (1-p)] v^{1-\beta }w\langle \nabla v , \nabla \eta \rangle \nonumber \\&+ 2 \gamma (1-p) v^{2-\beta } \frac{|\nabla \eta |^2}{\eta } w + \gamma v^{1-\beta } w {\mathscr {L}} [\eta ]\nonumber \\&+ \gamma v^{1-\beta }\eta w \left[ \frac{ \beta \Sigma (t,x,v)}{v}- 2 \Sigma _v(t,x,v) \right] \nonumber \\&- 2 \gamma v^{1-\beta } \eta \frac{\langle \nabla v, \Sigma _x(t,x,v) \rangle }{v^\beta }. \end{aligned}$$

Now the task is to provide suitable upper bounds for each of the terms on the right-hand side of (4.8). Towards this end we set $M= \sup _{Q_{R,T}} v$. Then starting from the first line, by recalling $0 \le \eta \le 1$ and using Young’s inequality we can write

4.9 $$\begin{aligned} 2 \gamma [(1-p)(m-1) k&v^{2-\beta } + h v^{1-\beta }]\eta w \le 2 \gamma [(1-p)(m-1) k v^{2-\beta } + h v^{1-\beta }] \eta ^{1/2} w\nonumber \\&\le \frac{1}{6} \eta w^2 + C \gamma ^2 [(1-p)^2 (m-1)^2 k^2 v^{4-2\beta } + h^2 v^{2-2\beta }] \nonumber \\&\le \frac{1}{6} \eta w^2 + C \gamma ^2 [(1-p)^2 (m-1)^2 k^2(\sup _{Q_{R, T}} v)^{4-2\beta } + h^2 (\sup _{Q_{R, T}} v)^{2-2\beta }] \nonumber \\&\le \frac{1}{6} \eta w^2 + C \gamma ^2 [(1-p)^2 (m-1)^2 k^2 M^{4-2\beta } + h^2 M^{2-2\beta }]. \end{aligned}$$

Note that here $\beta >0$ and we do restrict $\beta \le 1$. In particular $4- 2\beta \ge 2$ and $2- 2\beta \ge 0$ and so making use of the upper bound *M* of $v>0$ on $Q_{R,T}$ in (4.9) is justified. Moving next to the term on the second line of (4.8), by using the Cauchy-Schwarz inequality, recalling the relation $w = |\nabla v|^2/v^{\beta }$ and utilising (*iv*) in Lemma 4.1, we have

4.10 $$\begin{aligned}&2 \gamma [1-\beta (1-p)] v^{1-\beta }w \langle \nabla v , \nabla \eta \rangle \nonumber \\&\quad \le 2 \gamma |1-\beta (1-p)| v^{1-\beta }w |\nabla v| |\nabla \eta |\nonumber \\&\quad \le 2 \gamma |1-\beta (1-p)| v^{1-\beta /2} \eta ^{3/4} w^{3/2} \frac{|\nabla \eta |}{\eta ^{3/4}}\nonumber \\&\quad \le \frac{1}{6} (\eta ^{3/4} w^{3/2})^{4/3} + C \gamma ^4 [1-\beta (1-p)]^4 \left[ \frac{|\nabla \eta |}{\eta ^{3/4}}\right] ^4 v^{4-2\beta }\nonumber \\&\quad \le \frac{1}{6} \eta w^2 + \frac{C \gamma ^4 [1-\beta (1-p)]^4}{R^4} (\sup _{Q_{R, T}} v)^{4-2\beta }\nonumber \\&\quad \le \frac{1}{6} \eta w^2 + \frac{C \gamma ^4 [1-\beta (1-p)]^4}{R^4} M^{4-2\beta }. \end{aligned}$$

In much the same way for the third term in (4.8), by using Young’s inequality and taking advantage of (*iv*) in Lemma 4.1 we can write

4.11 $$\begin{aligned} 2 \gamma (1-p) v^{2-\beta } \frac{|\nabla \eta |^2}{\eta } w&\le \frac{1}{6} \eta w^2 +C \gamma ^2 (1-p)^2 \left[ \frac{|\nabla \eta |}{\eta ^{3/4}} \right] ^4 v^{4-2\beta }\nonumber \\&\le \frac{1}{6} \eta w^2 + \frac{C \gamma ^2 (1-p)^2}{R^4}(\sup _{Q_{R, T}} v)^{4-2\beta }\nonumber \\&\le \frac{1}{6} \eta w^2 + \frac{C \gamma ^2 (1-p)^2}{R^4} M^{4-2\beta }. \end{aligned}$$

We now come to the term $\gamma v^{1-\beta } w {\mathscr {L}} [\eta ] = \gamma v^{1-\beta } w[\partial _t - (1-p)v \Delta _f] \eta$ which has a purely geometric nature. We make use of the curvature bound ${{\mathscr {R}}ic}_f^m(g) \ge -(m-1)k g$ and the metric time-derivative bound $\partial _t g \ge -2hg$ as given in the theorem to give an upper bound. In fact, to handle this term more efficiently we consider the space and time derivatives separately.

- The term $\textbf{I} = \gamma (1-p) v^{2-\beta } w (-\Delta _f \eta )$: By virtue of ${{\mathscr {R}}ic}_f^m(g) \ge - (m-1)k g$ and the weighted Laplace comparison theorem we have $\Delta _f \varrho \le (m-1) \sqrt{k}\coth (\sqrt{k} \varrho )$ and so from (4.2) and the calculations leading to (4.3) we have
  4.12 $$\begin{aligned} \Delta _f \eta = {\bar{\eta }}_{\varrho \varrho }|\nabla \varrho |^2 + {\bar{\eta }}_\varrho \Delta _f \varrho \ge {\bar{\eta }}_{\varrho \varrho } + {\bar{\eta }}_\varrho (m-1)\sqrt{k} \coth (\sqrt{k} \varrho ). \end{aligned}$$
  Now using the bound $\sqrt{k} \coth (\sqrt{k} \varrho )\le \sqrt{k} \coth (\sqrt{k} R/2) \le (2+\sqrt{k} R)/R$ for $0 \le \varrho \le R/2$ and noting that ${\bar{\eta }}_\varrho = 0$ for $0 \le \varrho \le R/2$, this gives
  4.13 $$\begin{aligned} -\Delta _f \eta&\le - [ {\bar{\eta }}_{\varrho \varrho } + {\bar{\eta }}_\varrho (m-1)\sqrt{k} \coth (\sqrt{k} R/2)]\nonumber \\&\le |{\bar{\eta }}_{\varrho \varrho }| + (m-1)\left( \frac{2}{R} +\sqrt{k}\right) |{\bar{\eta }}_\varrho |. \end{aligned}$$
  Therefore substituting the above bound in the expression for $\textbf{I}$ gives
  4.14 $$\begin{aligned} \textbf{I}&=\gamma (1-p) v^{2-\beta } w (-\Delta _f \eta ) \nonumber \\&\le \gamma (1-p) v^{2-\beta } \sqrt{\eta }w \left[ \frac{|{\bar{\eta }}_{\varrho \varrho }|}{\sqrt{\eta }} +(m-1)\left( \frac{2}{R} +\sqrt{k} \right) \frac{|{\bar{\eta }}_\varrho |}{\sqrt{\eta }} \right] , \end{aligned}$$
  and so using Young’s inequality and (*iv*) in Lemma 4.1 it follows that
  4.15 $$\begin{aligned} \textbf{I}&\le \frac{\eta w^2}{12} + C\gamma ^2 (1-p)^2 \left[ \left( \frac{|{\bar{\eta }}_{\varrho \varrho }|}{\sqrt{\eta }}\right) ^2 +(m-1)^2\left( \frac{1}{R^2} +k \right) \left( \frac{|{\bar{\eta }}_\varrho |}{\sqrt{\eta }}\right) ^2 \right] v^{4-2\beta }\nonumber \\&\le \frac{\eta w^2}{12} + C \gamma ^2(1-p)^2(m -1)^2 \left[ \frac{1+ k R^2}{R^4}\right] (\sup _{Q_{R, T}} v)^{4-2\beta }. \end{aligned}$$
- The term $\textbf{II} = \gamma v^{1-\beta }w \partial _t \eta$: Here we use the bound $\partial _t \varrho (x,t) \ge - h R$ in $Q_{R,T}$ and Lemma 4.1. Hence noting $\partial _t \eta = \bar{\eta }_t + \bar{\eta }_\varrho \partial _t \varrho$ and (*iii*) in Lemma 4.1 we have
  4.16 $$\begin{aligned} \partial _t \eta&= \bar{\eta }_t + \bar{\eta }_\varrho \partial _t \varrho \le |\bar{\eta }_t| - h R \bar{\eta }_\varrho = |\bar{\eta }_t| + h R |\bar{\eta }_\varrho | \nonumber \\&\le C \left[ \frac{1}{\tau -t_0+T} + h \right] \sqrt{\eta }. \end{aligned}$$
  Hence substituting in the expression for $\textbf{II}$ and using Young’s inequality we can write
  4.17 $$\begin{aligned} \textbf{II}&= \gamma v^{1-\beta }w \partial _t \eta = \gamma \sqrt{\eta }v^{1-\beta } w \frac{\partial _t \eta }{\sqrt{\eta }} \le C \gamma \sqrt{\eta }v^{1-\beta } w \left[ \frac{1}{\tau -t_0+T} + h \right] \nonumber \\&\le \frac{1}{12} \eta w^2 + C \gamma ^2 \left[ \frac{1}{(\tau -t_0+T)^2} + h^2\right] (\sup _{Q_{R, T}} v)^{2-2\beta }. \end{aligned}$$

Therefore by putting together the two conclusions above for $\textbf{I}$ and $\textbf{II}$ we arrive at the bound

4.18 $$\begin{aligned} \gamma v^{1-\beta } w {\mathscr {L}} [\eta ] =&~\overbrace{\gamma (1-p) v^{2-\beta } w (-\Delta _f \eta )}^{\textbf{I}} + \overbrace{\gamma v^{1-\beta }w \partial _t \eta }^{\textbf{II}} \nonumber \\ \le&~ \frac{1}{6} \eta w^2 + C \gamma ^2 \left[ \frac{1}{(\tau -t_0+T)^2} + h^2\right] (\sup _{Q_{R, T}} v)^{2-2\beta }\nonumber \\&+ C \gamma ^2(1-p)^2 (m-1)^2\left[ \frac{1+ k R^2}{R^4}\right] (\sup _{Q_{R, T}} v)^{4-2\beta }\nonumber \\ \le&~ \frac{1}{6} \eta w^2 + C \gamma ^2 \left\{ \left[ \frac{1}{(\tau -t_0+T)^2} + h^2\right] M^{2-2\beta } \right. \nonumber \\&+ \left. (1-p)^2 (m-1)^2\left[ \frac{1+ k R^2}{R^4}\right] M^{4-2\beta } \right\} . \end{aligned}$$

Finally for the two remaining $\Sigma (t,x,v)$ terms, upon recalling $0\le \eta \le 1$ and utilising Young’s inequality we have

4.19 $$\begin{aligned} \gamma v^{1-\beta }\eta w&\bigg [\frac{\beta \Sigma (t,x,v)}{v}-2 \Sigma _v(t,x,v) \bigg ] \le \gamma v^{1-\beta }\sqrt{\eta }w \bigg [\frac{\beta \Sigma (t,x,v)}{v}-2 \Sigma _v(t,x,v) \bigg ]_+ \nonumber \\&\le \frac{1}{6} \eta w^2 + C \gamma ^2 \sup _{Q_{R, T}} \left\{ v^{2(1-\beta )} \left[ \frac{ \beta \Sigma (t,x,v)}{v}- 2 \Sigma _v(t,x,v) \right] _+^2\right\} , \end{aligned}$$

and likewise, by using the Cauchy-Schwarz and Young inequalities in turn we can write

4.20 $$\begin{aligned}&-2 \gamma v^{1-\beta } \eta \frac{\langle \nabla v, \Sigma _x(t,x,v) \rangle }{v^\beta } \nonumber \\&\quad \le ~2 \gamma v^{1-\beta } \eta |\nabla v| \frac{|\Sigma _x(t,x,v)|}{v^\beta } \le 2 \gamma \eta ^{1/4} w^{1/2} v^{1- \beta /2} \frac{|\Sigma _x(t,x,v)|}{v^\beta } \nonumber \\&\quad \le ~ \frac{1}{6} \eta w^2+C \gamma ^{4/3} \sup _{Q_{R, T}} \left\{ v^{(4/3)[1-\beta /2]} \left[ \frac{|\Sigma _x(t,x,v)|}{v^\beta }\right] ^{4/3}\right\} . \end{aligned}$$

Substituting all the above bounds in (4.8), rearranging terms and making note of $0 < \beta \le 1$ it follows that

4.21 $$\begin{aligned} \eta w^2 \le&~C(\gamma ) \left\{ \left( \begin{array}{ll} (1-p)^2 (m-1)^2\left[ \dfrac{1+ k R^2}{R^4}\right] +\dfrac{(1-p)^2}{R^4} \\ \\ +\dfrac{ [1-\beta (1-p)]^4}{R^4} +(1-p)^2 (m-1)^2 k^2 \end{array} \right) \right. M^{4-2\beta } \nonumber \\&\qquad + \left. \left( \begin{array}{ll} \left[ \dfrac{1}{(\tau -t_0+T)^2}+ 2h^2 \right] M^{2-2\beta } + \sup _{Q_{R, T}} \left\{ \left[ \dfrac{|\Sigma _x(t,x,v)|}{v^{(3\beta -2)/2}}\right] ^{4/3}\right\} \\ \\ +\sup _{Q_{R, T}} \left\{ v^{2(1-\beta )}\left[ \dfrac{\beta \Sigma (t,x,v)}{v}- 2 \Sigma _v(t,x,v) \right] _+^2\right\} \end{array} \right) \right\} . \end{aligned}$$

Recalling the maximality of $\eta w$ at $(x_1, t_1)$ along with $\eta \equiv 1$ when $d(x, x_0,t) \le R/2$ and $\tau \le t \le t_0$, it follows that $w^2 (x, \tau ) = (\eta ^2 w^2)(x, \tau ) \le (\eta ^2 w^2)(x_1,t_1) \le (\eta w^2)(x_1,t_1)$. Hence noting $w = |\nabla v|^2/v^{\beta }$ and absorbing $\beta$, *p* and *m* into $C(\gamma )$ gives

4.22 $$\begin{aligned} \frac{|\nabla v|}{v^{\beta /2}} (x, \tau ) \le C(p, \beta , m) \left\{ \begin{array}{ll} \left[ \dfrac{k^{1/4}}{\sqrt{R}}+\dfrac{1}{R}+\sqrt{k} \right] M^{1-\beta /2} + \sqrt{h} M^{(1-\beta )/2} \\ \\ + \dfrac{M^{(1-\beta )/2}}{\sqrt{\tau -t_0+T}} + \sup _{Q_{R, T}} \left\{ \left[ \dfrac{|\Sigma _x(t,x,v)|}{v^{(3\beta -2)/2}}\right] ^{1/3}\right\} \\ \\ + \sup _{Q_{R, T}} \left\{ v^{(1-\beta )/2} \left[ \dfrac{\beta \Sigma (t,x,v)}{v}- 2 \Sigma _v(t,x,v) \right] _+^{1/2}\right\} \end{array} \right\} . \end{aligned}$$

The arbitrariness of $\tau$ in the interval $(t_0-T, t_0]$ now gives the desired conclusion. $\square$

## Ancient solutions to $$\partial _t u - \Delta _f u^p = {{\mathscr {N}}}(u)$$$$\textbf{I}$$: proof of Theorem 2.5

Let us now attend to the proof of Theorem 2.5. Towards this end fix a space-time point $(x_0,t_0)$ and set $t=t_0$, $R>0$ and $T=R^2$. It then follows from the local estimate (2.4) in Theorem 2.4 (with $k=0$) that

5.1 $$\begin{aligned} \frac{|\nabla v|}{v^{\beta /2}}(x_0,t_0)&\le C \left\{ \begin{array}{ll} \dfrac{M^{1-\beta /2}}{R} + \dfrac{M^{(1-\beta )/2}}{\sqrt{T}} + \sup _{Q_{R, T}} \left\{ \left[ \dfrac{|\Sigma _x(t,x,v)|}{v^{(3\beta -2)/2}}\right] ^{1/3}\right\} \\ \\ + \sup _{Q_{R, T}}\left\{ v^{(1-\beta )/2} \left[ \dfrac{ \beta \Sigma (t,x,v)}{v} - 2 \Sigma _v(t,x,v) \right] _+^{1/2}\right\} \end{array} \right\} . \end{aligned}$$

By Remark 2.2 and (2.5) we have $\Sigma (t,x,v) = p u^{p-2} {{\mathscr {N}}}(u)$ with $u=[(1-p)v/p]^{1/(p-1)}$. Hence $\Sigma$ does not depend explicitly on *t* and *x* and so in particular $\Sigma _x \equiv 0$. Moreover by writing $\Sigma _v=\Sigma _u \partial _v u$ where $\Sigma _u = pu^{p-2}[(p-2){{\mathscr {N}}}/u+{{\mathscr {N}}}_u]$ and $\partial _v u = -u^{2-p}/p$ we have $\Sigma _v=(2-p){\mathscr {N}}/u-{{\mathscr {N}}}_u$. There using the assumptions in the theorem

5.2 $$\begin{aligned} \beta \frac{\Sigma (v)}{v} - 2 \Sigma _v (v)&= \beta (1-p) \frac{{{\mathscr {N}}}(u)}{u} - 2 \left[ (2-p) \frac{{{\mathscr {N}}}(u)}{u} - {{\mathscr {N}}}_u(u) \right] \nonumber \\&= [\beta (1-p)-2(2-p)] \frac{{{\mathscr {N}}}(u)}{u} + 2 {\mathscr {N}}_u(u) \le 0, \end{aligned}$$

and so $[\beta \Sigma /v-2\Sigma _v]_+\equiv 0$. Now from the stated growth assumption in the theorem $1/u(x,t) = o([\varrho (x) + \sqrt{|t|}]^{2/[(1-p)(2-\beta )]})$ we obtain

5.3 $$\begin{aligned} v = [p/(1-p)] u^{p-1} = o([\varrho (x) + \sqrt{|t|}]^{2/(2-\beta )}), \end{aligned}$$

and as a result $M = \sup _{Q_{R,T}} v = o(R^{2/(2-\beta )})$. Therefore from the above and (5.1) we conclude that

5.4 $$\begin{aligned} \frac{|\nabla v|}{v^{\beta /2}}(x_0,t_0) \le C \left[ \dfrac{M^{1-\beta /2}}{R} + \dfrac{M^{(1-\beta )/2}}{\sqrt{T}} \right] \le \frac{o(R)}{R} + \frac{o(R^{(1-\beta )/(2-\beta )})}{R}. \end{aligned}$$

Now passing to the limit $R \nearrow \infty$ it follows that $|\nabla v|(x_0,t_0)=0$. The arbitrariness of $(x_0,t_0)$ implies $|\nabla v| \equiv 0$ and so *v* and subsequently *u* are spatially constant. Hence we have $u=u(t)$. From the equation satisfied by *u* it then follows that $du/dt = {{\mathscr {N}}}(u)$. Assuming now that ${{\mathscr {N}}}(u) \ge a >0$ for all $u>0$ it then follows by integrating the ODE that $u(t) \le u(0) + at$ for all $t<0$. This however clashes with $u(t)>0$ as $t \searrow -\infty$ and so the conclusion is reached. $\square$

## The Ricci-Perelman super flow $\textbf{I}$:$\partial _t g + 2(1-p) v {{\mathscr {R}}ic}^m_f(g) \ge -2\textsf{k}g$

In this section we obtain further global gradient bounds on positive smooth solutions to (1.1) in the *super-critical* range when *M* is closed and the metric and potential evolve under the super flow [*cf.* (1.11) and (1.12)]

6.1 $$\begin{aligned} \frac{1}{2} \dfrac{\partial g}{\partial t} (x,t) + (1-p) v(x,t) {{\mathscr {R}}ic}^m_f(g)(x,t) \ge - \textsf{k}g(x,t), \qquad {\mathsf k} \ge 0. \end{aligned}$$

### Lemma 6.1

Let u be a positive solution to (1.1) and set $v =p/(1-p) u^{p-1}$ where $0<p<1$. For $s \ge 2$, $q \in {\mathbb {R}}$, $\zeta =\zeta (t)$ non-negative and of class $\mathscr {C}^1[0, \infty )$ let

6.2 $$\begin{aligned} {{\textsf{H}}}^{s,q}_\zeta [v] = \zeta (t) \frac{|\nabla v|^s}{v^q} + \Gamma (v). \end{aligned}$$

Here $\Gamma =\Gamma (v)$ is a function of class $\mathscr {C}^2(0, \infty )$. Then ${\textsf{H}} = {{\textsf{H}}}^{s, q}_\zeta [v]$ satisfies the evolution identity,

6.3 $$\begin{aligned} {{\mathscr {L}}} ({{\textsf{H}}}^{s,q}_\zeta [v]) =&~ \zeta '(t) \frac{|\nabla v|^s}{v^q} - s \zeta (t) \frac{|\nabla v|^{s-2}}{v^q} \left[ \frac{1}{2} \partial _t g + (1-p) v {{\mathscr {R}}ic}_f^m(g) \right] ( \nabla v, \nabla v) \nonumber \\&+s(1-p)\zeta (t) \frac{|\nabla v|^{s-2}}{v^q} \left[ |\nabla v|^2 \Delta _f v - v |\nabla \nabla v|^2 - v \frac{\langle \nabla f , \nabla v \rangle ^2}{(m-n)} \right] \nonumber \\&+ s [q(1-p)-1] \zeta (t) \langle \nabla |\nabla v|^2 , \nabla v\rangle \frac{|\nabla v|^{s-2}}{v^{q}} -q[q(1-p) -p] \zeta (t) \frac{|\nabla v|^{s+2}}{v^{q+1}}\nonumber \\&-\frac{s(1-p)}{2v^{q-1}} \zeta (t) \langle \nabla |\nabla v|^{s-2} , \nabla |\nabla v|^2 \rangle - s \zeta (t) \frac{|\nabla v|^{s-2}}{v^q}\langle \nabla v, \nabla \Sigma (t,x,v) \rangle \nonumber \\&+ q \zeta (t)\frac{|\nabla v|^s}{v^{q+1}} \Sigma (t,x,v) - \Gamma '(v) \Sigma (t,x,v) - [\Gamma '(v) + (1-p)v\Gamma ''(v)] |\nabla v|^2. \end{aligned}$$

### Proof

Referring to (6.2) it is seen in view of linearity that

6.4 $$\begin{aligned} {{\mathscr {L}}} ({{\textsf{H}}}^{s,q}_\zeta [v]) = \zeta (t) {{\mathscr {L}}} \left[ \frac{|\nabla v|^s}{v^q}\right] + \zeta '(t) \frac{|\nabla v|^s}{v^q} + {{\mathscr {L}}} [\Gamma (v)]. \end{aligned}$$

We need to evaluate the expressions in the first and third terms on the right-hand side respectively. Focusing on the first term, it is easily seen that,

6.5 $$\begin{aligned} {{\mathscr {L}}} \left[ \frac{|\nabla v|^s}{v^q} \right] =&~ \frac{1}{v^q} {{\mathscr {L}}} [|\nabla v|^s] + 2 (1-p) v \left\langle \nabla \left( \frac{|\nabla v|^s}{v^q}\right) , \frac{\nabla v^q}{v^q} \right\rangle - \frac{|\nabla v|^s}{v^{2q}} {{\mathscr {L}}} [v^q]. \end{aligned}$$

Now regarding the first term on the right-hand side of (6.5), a direct calculation gives

6.6 $$\begin{aligned} {{\mathscr {L}}} [|\nabla v|^s] =&~\frac{s}{2} |\nabla v|^{s-2} \left[ \partial _t -(1-p)v \Delta _f\right] |\nabla v|^2 - \frac{s}{2}(1-p)v \langle \nabla |\nabla v|^{s-2} , \nabla |\nabla v|^2 \rangle \nonumber \\ =&~ \frac{s}{2} |\nabla v|^{s-2} {{\mathscr {L}}} [|\nabla v|^2] - \frac{s}{2}(1-p)v \langle \nabla |\nabla v|^{s-2} , \nabla |\nabla v|^2 \rangle , \end{aligned}$$

whilst using Lemma 3.1 and the weighted Bochner-Weitzenböck formula (1.5) we have

6.7 $$\begin{aligned} {{\mathscr {L}}} [|\nabla v|^2] =&-[\partial _t g] ( \nabla v, \nabla v) +2 (1-p) |\nabla v|^2 \Delta _f v - 2\langle \nabla v, \nabla |\nabla v|^2 \rangle \nonumber \\&-2 \langle \nabla v, \nabla \Sigma (t,x,v) \rangle -2(1-p) v |\nabla \nabla v|^2\nonumber \\&-2(1-p)v {{\mathscr {R}}ic}_f^m (\nabla v, \nabla v) - 2 (1-p) v \langle \nabla f , \nabla v \rangle ^2/(m-n). \end{aligned}$$

Therefore by combining the two identities above it follows that

6.8 $$\begin{aligned} {{\mathscr {L}}} [|\nabla v|^s] =&-\frac{s}{2} |\nabla v|^{s-2} [\partial _t g] ( \nabla v, \nabla v) + s (1-p) |\nabla v|^s \Delta _f v - s |\nabla v|^{s-2}\langle \nabla v, \nabla |\nabla v|^2 \rangle \nonumber \\&- s |\nabla v|^{s-2}\langle \nabla v, \nabla \Sigma (t,x,v) \rangle - s (1-p) v |\nabla v|^{s-2} |\nabla \nabla v|^2\nonumber \\&- s (1-p)v |\nabla v|^{s-2} {{\mathscr {R}}ic}_f^m (\nabla v, \nabla v) -s (1-p)v |\nabla v|^{s-2} \langle \nabla f , \nabla v \rangle ^2/(m-n)\nonumber \\&-\frac{s}{2}(1-p)v \langle \nabla |\nabla v|^{s-2} , \nabla |\nabla v|^2 \rangle . \end{aligned}$$

Next, for the second term on the right-hand side of (6.5) we can write

6.9 $$\begin{aligned} \left\langle \nabla \left( \frac{|\nabla v|^s}{v^q}\right) , \frac{\nabla v^q}{v^q} \right\rangle = \frac{sq}{2} \frac{|\nabla v|^{s-2}}{v^{q+1}}\langle \nabla |\nabla v|^2 , \nabla v\rangle - q^2 \frac{|\nabla v|^{s+2}}{v^{q+2}}, \end{aligned}$$

and in a similar way for the third term on the right-hand side of (6.5) we have

6.10 $$\begin{aligned} {{\mathscr {L}}} [v^q]&= q v^{q-1} [\partial _t - (1-p) v \Delta _f] v - q(1-p)(q-1) v^{q-1} |\nabla v|^2\nonumber \\&=-q v^{q-1} |\nabla v|^2- q v^{q-1}\Sigma (t,x,v) - q (1-p)(q-1) v^{q-1} |\nabla v|^2 \nonumber \\&=-q v^{q-1}\Sigma (t,x,v) - q [q(1-p)+p] v^{q-1} |\nabla v|^2. \end{aligned}$$

Substituting (6.8), (6.9) and (6.10) back into (6.5) and rearranging terms leads to

6.11 $$\begin{aligned} {{\mathscr {L}}} \left[ \frac{|\nabla v|^s}{v^q} \right] =&-s\frac{|\nabla v|^{s-2}}{v^q} \left[ \frac{1}{2}\partial _t g+(1-p) v {{\mathscr {R}}ic}_f^m(g)\right] ( \nabla v, \nabla v) \nonumber \\&+ s (1-p) \frac{|\nabla v|^{s-2}}{v^q} \left[ |\nabla v|^2 \Delta _f v - v |\nabla \nabla v|^2 - v \frac{\langle \nabla f , \nabla v \rangle ^2}{(m-n)} \right] \nonumber \\&- s[1-q(1-p)] \langle \nabla |\nabla v|^2 , \nabla v\rangle \frac{|\nabla v|^{s-2}}{v^{q}} - q[q(1-p) -p] \frac{|\nabla v|^{s+2}}{v^{q+1}}\nonumber \\&-\frac{s(1-p)}{2v^{q-1}} \langle \nabla |\nabla v|^{s-2} , \nabla |\nabla v|^2 \rangle - s \frac{|\nabla v|^{s-2}}{v^q}\langle \nabla v, \nabla \Sigma (t,x,v) \rangle \nonumber \\&+ \frac{q|\nabla v|^s}{v^{q+1}} \Sigma (t,x,v). \end{aligned}$$

This completes the calculation of the first term on the right-hand side of (6.4). Since for the third term of the same equation we can write

6.12 $$\begin{aligned} {{\mathscr {L}}} (\Gamma (v))&= \Gamma '(v) [-|\nabla v|^2 - \Sigma (t,x,v)] - (1-p)v\Gamma ''(v) |\nabla v|^2, \end{aligned}$$

the conclusion follows upon substituting (6.11) and (6.12) back into (6.4). $\square$

### Lemma 6.2

Under the assumptions of Lemma 6.1, if the metric and potential evolve under the super flow (6.1) then ${\textsf{H}} = {{\textsf{H}}}^{s, q}_\zeta [v]$ satisfies the evolution inequality

6.13 $$\begin{aligned} {{\mathscr {L}}} ({{\textsf{H}}}^{s,q}_\zeta [v]) - 2&[q(1-p)-1] \langle \nabla v, \nabla {{\textsf{H}}}^{s,q}_\zeta [v]\rangle \nonumber \\ \le&~ \frac{|\nabla v|^s}{v^q} \left\{ \zeta '(t) + s {{\textsf{k}}} \zeta (t) + \zeta (t) \left[ q \frac{\Sigma (t,x,v)}{v} - s \Sigma _v(t,x,v)] \right] \right\} \nonumber \\&+ \zeta (t) (1-p) \left[ q^2 - \frac{2-p}{1-p} q+\frac{sm}{4} \right] \frac{|\nabla v|^{s+2}}{v^{q+1}}\nonumber \\&- s \zeta (t) \frac{|\nabla v|^{s-2}}{v^q}\langle \nabla v, \Sigma _x (t,x,v) \rangle + \Gamma '(v) \Sigma (t,x,v) \nonumber \\&- (1-p) \left[ \frac{2q(1-p)-1}{1-p} \Gamma '(v) + v\Gamma ''(v) \right] |\nabla v|^2. \end{aligned}$$

### Proof

Referring to the second line in the right-hand side of (6.3) and making note of the inequality $|\nabla \nabla v|^2 + \langle \nabla f, \nabla v \rangle ^2/(m-n) \ge (\Delta _f v)^2/m$, we can write

6.14 $$\begin{aligned} \frac{|\nabla v|^{s-2}}{v^q}&\left[ |\nabla v|^2 \Delta _f v -v|\nabla \nabla v|^2 - \frac{v\langle \nabla f , \nabla v \rangle ^2}{m-n}\right] \nonumber \\&\le \frac{|\nabla v|^{s-2}}{v^{q-1}} \left[ \frac{|\nabla v|^2 \Delta _f v}{v} - \frac{(\Delta _f v)^2}{m}\right] \nonumber \\&\le \frac{|\nabla v|^{s-2}}{v^{q-1}} \left[ \frac{m}{4} \frac{|\nabla v|^4}{v^2} -\left( \frac{\sqrt{m}}{2} \frac{|\nabla v|^2}{v} - \frac{\Delta _f v}{\sqrt{m}} \right) ^2\right] \le \frac{m}{4} \frac{|\nabla v|^{s+2}}{v^{q+1}}. \end{aligned}$$

Therefore substituting the above inequality in (6.3) results in

6.15 $$\begin{aligned} {{\mathscr {L}}} ({{\textsf{H}}}^{s,q}_\zeta [v]) \le&~ \zeta '(t) \frac{|\nabla v|^s}{v^q} - s \zeta (t) \frac{|\nabla v|^{s-2}}{v^q} \left[ \frac{1}{2}\partial _t g+(1-p)v{{\mathscr {R}}ic}_f^m(g)\right] ( \nabla v, \nabla v) \nonumber \\&+ \frac{s(1-p)m}{4} \zeta (t) \frac{|\nabla v|^{s+2}}{v^{q+1}} + s[q(1-p)-1] \zeta (t) \langle \nabla |\nabla v|^2 , \nabla v\rangle \frac{|\nabla v|^{s-2}}{v^{q}}\nonumber \\&- q[q(1-p) -p] \zeta (t) \frac{|\nabla v|^{s+2}}{v^{q+1}} - \frac{s(1-p)}{2v^{q-1}} \zeta (t) \langle \nabla |\nabla v|^{s-2} , \nabla |\nabla v|^2 \rangle \nonumber \\&- s\zeta (t) \frac{|\nabla v|^{s-2}}{v^q}\langle \nabla v, \nabla \Sigma (t,x,v) \rangle + \frac{q \zeta (t)}{v^{q+1}} |\nabla v|^s\Sigma (t,x,v)\nonumber \\&-\Gamma '(v) \Sigma (t,x,v) - [\Gamma '(v) + (1-p)v\Gamma ''(v)]|\nabla v|^2. \end{aligned}$$

Now by using the following identity

6.16 $$\begin{aligned} \left\langle \nabla v, \nabla \left( \frac{|\nabla v|^s}{v^q}\right) \right\rangle = \frac{s}{2} \frac{|\nabla v|^{s-2}}{v^q} \langle \nabla v, \nabla |\nabla v|^2 \rangle - q \frac{|\nabla v|^{s+2}}{v^{q+1}}, \end{aligned}$$

and substituting back in (6.15) we can write

6.17 $$\begin{aligned} {{\mathscr {L}}} ({{\textsf{H}}}^{s,q}_\zeta [v]) \le&~ \zeta '(t) \frac{|\nabla v|^s}{v^q} - s \zeta (t) \frac{|\nabla v|^{s-2}}{v^q} \left[ \frac{1}{2}\partial _t g+(1-p) v {{\mathscr {R}}ic}_f^m(g)\right] ( \nabla v, \nabla v) \nonumber \\&+\zeta (t) \frac{s(1-p)m}{4} \frac{|\nabla v|^{s+2}}{v^{q+1}} + \zeta (t)[(1-p)q^2 - (2-p)q] \frac{|\nabla v|^{s+2}}{v^{q+1}}\nonumber \\&+ 2 \zeta (t) [q(1-p)-1] \langle \nabla v, \nabla (|\nabla v|^s/v^q) \rangle + \frac{q \zeta (t)}{v^{q+1}} |\nabla v|^s\Sigma (t,x,v)\nonumber \\&- \zeta (t)\frac{s(1-p)}{2v^{q-1}} \langle \nabla |\nabla v|^{s-2} , \nabla |\nabla v|^2 \rangle - s\zeta (t) \frac{|\nabla v|^{s-2}}{v^q}\langle \nabla v, \nabla \Sigma (t,x,v) \rangle \nonumber \\&- \Gamma '(v) \Sigma (t,x,v) - [\Gamma '(v) + (1-p)v\Gamma ''(v)] |\nabla v|^2. \end{aligned}$$

Next rearranging terms and using the super flow inequality (6.1) leads to

6.18 $$\begin{aligned} {{\mathscr {L}}} ({{\textsf{H}}}^{s,q}_\zeta [v]) \le&~ \zeta '(t) \frac{|\nabla v|^s}{v^q} + s {\textsf{k}} \zeta (t) \frac{|\nabla v|^{s}}{v^q} +\zeta (t)(1-p) \left[ q^2 - \frac{2-p}{1-p} q+\frac{sm}{4} \right] \frac{|\nabla v|^{s+2}}{v^{q+1}} \nonumber \\&+ q\zeta (t) \frac{|\nabla v|^s}{v^q}\frac{\Sigma (t,x,v)}{v} + 2 \zeta (t) [q(1-p)-1] \langle \nabla v, \nabla (|\nabla v|^s/v^q) \rangle \nonumber \\&- s\zeta (t) \frac{|\nabla v|^{s-2}}{v^q}\langle \nabla v, \nabla \Sigma (t,x,v) \rangle - \zeta (t)\frac{s(1-p)}{2v^{q-1}} \langle \nabla |\nabla v|^{s-2} , \nabla |\nabla v|^2 \rangle \nonumber \\&- \Gamma '(v) \Sigma (t,x,v) - [\Gamma '(v) + (1-p)v\Gamma ''(v)] |\nabla v|^2. \end{aligned}$$

Now referring to (6.2), by adding and subtracting suitable terms to the right-hand side of (6.18), we can rewrite this as

6.19 $$\begin{aligned} {{\mathscr {L}}} ({{\textsf{H}}}^{s,q}_\zeta [v]) \le&~ \zeta '(t) \frac{|\nabla v|^s}{v^q} + s {\textsf{k}} \zeta (t) \frac{|\nabla v|^{s}}{v^q} + 2 [q(1-p)-1] \langle \nabla v, \nabla \overbrace{[\zeta (t)|\nabla v|^s/v^q + \Gamma (v)]}^{{{\textsf{H}}}^{s,q}_\zeta [v]} \rangle \nonumber \\&+ q\zeta (t)\frac{|\nabla v|^s}{v^{q}}\frac{\Sigma (t,x,v)}{v} +\zeta (t)(1-p) \left[ q^2 - \frac{2-p}{1-p} q+\frac{sm}{4} \right] \frac{|\nabla v|^{s+2}}{v^{q+1}} \nonumber \\&- s\zeta (t)\frac{|\nabla v|^{s-2}}{v^q}\langle \nabla v, \nabla \Sigma (t,x,v) \rangle - \zeta (t)\frac{s(1-p)}{2v^{q-1}} \langle \nabla |\nabla v|^{s-2} , \nabla |\nabla v|^2 \rangle \nonumber \\&- 2 [q(1-p)-1]\Gamma '(v) |\nabla v|^2 - \Gamma '(v) \Sigma (t,x,v) \nonumber \\&- [\Gamma '(v)+(1-p)v\Gamma ''(v)] |\nabla v|^2. \end{aligned}$$

Finally rearranging terms, using (6.2), and $\langle \nabla v, \nabla \Sigma \rangle = \langle \nabla v, \Sigma _x \rangle + \Sigma _v |\nabla v|^2$ along with $\langle \nabla |\nabla v|^{s-2}, \nabla |\nabla v|^2\rangle \ge 0$ when $s \ge 2$ gives the desired conclusion. Note that the last inequality follows by writing $V=|\nabla v|^2$, setting $\alpha =(s-2)/2$ (recall $s\ge 2$) and observing that $\langle \nabla V^\alpha , \nabla V \rangle = \alpha V^{\alpha -1} |\nabla V|^2 \ge 0$. $\square$

### Remark 6.3

The quadratic polynomial $q^2-(2-p)/(1-p)q+sm/4$ appearing in (6.13) has non-negative discriminant when $1-1/[\sqrt{sm}-1]\le p<1$. If *q* is chosen between the roots $0<q_1\le q_2$ then this polynomial will be non-positive at *q*. Note also that in the closed case in corollaries below the left endpoint of the *p* interval *can* be admitted. In the general case, due to localisation and use of cut-off functions, we need the strict inequality.

### Corollary 6.4

Let $(M,g,e^{-f} dv_g)$ be a smooth metric measure space with *M* closed. Let *u* be a positive smooth solution to (1.1) where $0<1-1/[\sqrt{sm}-1]\le p<1$, $s \ge 2$ and set $v=p/(1-p) u^{p-1}$. Assume the metric and potential satisfy the super flow (6.1) with $\textsf{k} \ge 0$ and for some *q* between $q_1$ and $q_2$ and $a \in {\mathbb {R}}$ the following hold:

- $$\Gamma '(v) \Sigma (t,x,v) \le 0$$,
- $$\langle \nabla v, \Sigma _x(t,x,v) \rangle \ge 0$$,
- $$(q/s) \Sigma (t,x,v)/v - \Sigma _v(t,x,v) \le a$$,
- $$[2q(1-p)-1]\Gamma '(v)+(1-p)v\Gamma ''(v) \ge 0$$.

Then for all $x \in M$ and $0<t \le T$ we have

6.20 $$\begin{aligned} {[}|\nabla v|^s/v^q] (x,t) \le e^{s(\textsf{k}+a)t} \left\{ \max _{M} \left[ |\nabla v|^s/v^q + \Gamma (v) \right] _{t=0} - \Gamma (v(x,t)) \right\} . \end{aligned}$$

### Proof

Using (6.13) and the assumptions on $\Gamma$ and $\Sigma$ in the statement of the corollary, we can write for ${{\textsf{H}}} = {{\textsf{H}}}^{s,q}_\zeta [v] = \zeta (t) |\nabla v|^s/v^q + \Gamma (v)$

6.21 $$\begin{aligned} {{\mathscr {L}}} ({{\textsf{H}}}^{s,q}_\zeta [v])&- 2 [q(1-p)-1] \langle \nabla v, \nabla {{\textsf{H}}}^{s,q}_\zeta [v]\rangle \nonumber \\ \le&\left\{ \zeta '(t) + s {{\textsf{k}}} \zeta (t) + \zeta (t) \left[ q\frac{\Sigma (t,x,v)}{v} - s \Sigma _v(t,x,v) \right] \right\} \frac{|\nabla v|^s}{v^q} \nonumber \\&+\zeta (t) \left\{ (1-p) \left[ q^2 - \frac{2-p}{1-p} q+\frac{sm}{4} \right] \frac{|\nabla v|^{s+2}}{v^{q+1}} - s \frac{|\nabla v|^{s-2}}{v^q}\langle \nabla v, \Sigma _x (t,x,v) \rangle \right\} \nonumber \\&+ \Gamma '(v) \Sigma (t,x,v) - (1-p) \left[ \frac{2q(1-p)-1}{1-p} \Gamma '(v)+v\Gamma ''(v)\right] |\nabla v|^2 \nonumber \\ \le&~[\zeta '(t) + s ({{\textsf{k}}} + a) \zeta (t)] \frac{|\nabla v|^s}{v^q} +\zeta (t)(1-p) \left[ q^2 - \frac{2-p}{1-p} q+\frac{sm}{4} \right] \frac{|\nabla v|^{s+2}}{v^{q+1}}. \end{aligned}$$

The function $\zeta (t) = e^{-s(\textsf{k}+a)t}$ is non-negative, smooth and satisfies $\zeta '+s(\textsf{k}+a)\zeta =0$. Thus substituting in (6.21) and noting the range of *p* we have

6.22 $$\begin{aligned} {{\mathscr {L}}} ({{\textsf{H}}}^{s,q}_\zeta [v]) - 2 [q(1-p)-1] \langle \nabla v, \nabla {{\textsf{H}}}^{s,q}_\zeta [v]\rangle \le 0. \end{aligned}$$

The assertion is now a consequence of the weak maximum principle giving

6.23 $$\begin{aligned} {{\textsf{H}}}^{s,q}_\zeta [v](x,t)&= e^{-s(\textsf{k}+a)t} [|\nabla v|^s/v^q](x,t) + \Gamma (v(x,t)) \nonumber \\&\le \max _M [|\nabla v|^s/v^q + \Gamma (v)]_{t=0} = \max _M {\mathsf H}^{s,q}_\zeta [v](x,0). \end{aligned}$$

The proof is thus complete. $\square$

### Corollary 6.5

Let $(M,g,e^{-f} dv_g)$ be a smooth metric measure space with *M* closed. Let *u* be a positive smooth solution to (1.1), $0<p_c \le p<1$ and set $v=p/(1-p) u^{p-1}$. Assume the metric and potential satisfy the super flow (6.1) with $\textsf{k} \ge 0$ and for some $1<q<1/(1-p)$ the following hold:

- $$\Sigma (t,x,v) \ge 0$$,
- $$\langle \nabla v, \Sigma _x(t,x,v) \rangle \ge 0$$,
- $$2\Sigma _v(t,x,v) - q\Sigma (t,x,v)/v \ge 0$$.

Then for $x \in M$ and $0<t \le T$ we have

6.24 $$\begin{aligned} t \frac{|\nabla v|^2}{v^q}(x,t) \le \frac{1+2{\mathsf k}t}{(1-q)[(1-p)q-1]} \left[ \max _M v^{1-q}(x,0) - v^{1-q}(x,t) \right] . \end{aligned}$$

### Remark 6.6

Corollary 6.5 can be extended to the range $0<1-1/[\sqrt{2m}-1] \le p<p_c$ by requiring *q* to lie in $[q_1, q_2]=[q_1, q_2] \cap (1, 1/(1-p))$. For $p_c\le p<1$ it is easily seen that $(1,1/(1-p)) \subset (q_1, q_2)$ and so $(1,1/(1-p))=[q_1, q_2] \cap (1, 1/(1-p))$. In contrast in the sub-critical range $0<1-1/[\sqrt{2m}-1] \le p<p_c$ we have $[q_1, q_2] \subset (1,1/(1-p))$. Note that $p \ge p_c=1-2/m$ is equivalent to both conditions $q_1 \le 1$ and $1/(1-p) \le q_2$. See also Remark 6.3 regarding the reason for $p \ge 1-1/[\sqrt{2\,m}-1]$.

### Proof

For ${{\textsf{H}}}[v] = \zeta (t) |\nabla v|^2/v^q + cv^{1-q}$ where $s=2$, $\Gamma (v)=cv^{1-q}$ and $c>0$ is to be determined below, we can write using (6.13) in Lemma 6.2,

6.25 $$\begin{aligned} {{\mathscr {L}}} ({{\textsf{H}}}) - 2&[q(1-p)-1] \langle \nabla v, \nabla {{\textsf{H}}}\rangle \nonumber \\ \le&\left\{ \zeta '(t) + 2 {{\textsf{k}}} \zeta (t) + \zeta (t) \left[ q\frac{\Sigma (t,x,v)}{v} - 2 \Sigma _v(t,x,v) \right] \right\} \frac{|\nabla v|^2}{v^q} \nonumber \\&+\zeta (t)(1-p) \left[ q^2 - \frac{2-p}{1-p} q+\frac{m}{2} \right] \frac{|\nabla v|^4}{v^{q+1}} - 2\frac{\zeta (t)}{v^q}\langle \nabla v, \Sigma _x (t,x,v) \rangle \nonumber \\&- c(1-q) [q(1-p)-1] \frac{|\nabla v|^2}{v^q} + c(1-q) \frac{\Sigma (t,x,v)}{v^q}. \end{aligned}$$

Here we have made use of $\Gamma '(v) \Sigma (t,x,v)=c(1-q) \Sigma (t,x,v)/v^q$ and

6.26 $$\begin{aligned} (1-p) \left[ \frac{2q(1-p)-1}{1-p} \Gamma '(v)+v\Gamma ''(v)\right] = c(1-q) [q(1-p)-1] \frac{1}{v^q}. \end{aligned}$$

Setting $c=\{(1-q)[q(1-p)-1]\}^{-1}>0$ [note $1<q<1/(1-p)$], the assumptions give

$$\begin{aligned} \mathrm{RHS \, } (6.25) \le [\zeta '(t) + 2 {\textsf{k}} \zeta (t) -1] \frac{|\nabla v|^2}{v^q} +\zeta (t)(1-p) \left[ q^2 - \frac{2-p}{1-p} q+\frac{m}{2} \right] \frac{|\nabla v|^4}{v^{q+1}}. \end{aligned}$$

Now when $\textsf{k} \ge 0$ by taking $\zeta (t) = t/(1+2\textsf{k} t)$ we have $(\zeta ' + 2 \textsf{k} \zeta -1) \le 0$. Thus since $p \ge 1-1/[\sqrt{2\,m}-1]$ (*see* Remark 6.6) we obtain ${{\mathscr {L}}}({{\textsf{H}}}) - 2 [q(1-p)-1] \langle \nabla v, \nabla {{\textsf{H}}} \rangle \le 0$. The conclusion now follows by an application of the weak maximum principle. $\square$

## A Hamilton-Souplet-Zhang estimate in the range $p_0(m)< p < 1$

In this section we present another set of gradient estimates for positive solutions to (1.1). The range is $p_0< p<1$ where $p_0=p_0(m)>0$ is the larger of the values 1/2 and $1-1/\sqrt{m-1}$ ($m \ge 2)$, that is, $p_0=1/2$ when $2 \le m \le 5$ and $p_0=1-1/\sqrt{m-1}$ when $m \ge 5$. Thus we always have $p_0(m) \ge 1/2$, and when $m \ge 4$ we have $p_0 \le p_c=1-2/m$. Hence for $m \ge 4$, $(p_0,1)$ is larger than $(p_c,1)$ and extends into the sub-critical range. Throughout we use the notation

7.1 $$\begin{aligned} \Sigma ^\star (t, x, v) = (p -1/2) v^{1-\frac{1}{p-1/2}} {\mathscr {N}} (t,x,v^{\frac{1}{p-1/2}}). \end{aligned}$$

### Theorem 7.1

Let $(M, g, d\mu )$ be a complete smooth metric measure space with $d\mu =e^{-f} dv_g$. Suppose that the metric and potential are time dependent, of class $\mathscr {C}^2$ and that for suitable constants $k, h \ge 0$ and $m \ge n$ satisfy ${{\mathscr {R}}ic}_f^m (g) \ge -(m-1)kg$, $\partial _t g \ge -2\,h g$ in the compact space-time cylinder $Q_{R,T}$ with $R, T >0$. Let *u* be a positive solution to (1.1) with $p_0< p < 1$ and let $v = u^{p-1/2}$ and $M= \sup _{Q_{R,T}} v$. Then there exists $C=C(p,m)>0$ such that for every (*x*, *t*) in $Q_{R/2,T}$ with $t>t_0-T$ we have

7.2 $$\begin{aligned} |\nabla v|(x,t) \le C \left\{ \begin{array}{ll} \sqrt{h} M^\frac{p}{2p-1} + \left[ \dfrac{k^{1/4}}{\sqrt{R}} + \dfrac{1}{R} + \sqrt{k} \right] M \\ \\ +\sup _{Q_{R, T}} \left\{ v^{\frac{2p}{3(2p-1)}} |\Sigma ^\star _x(t,x,v)|^{1/3}\right\} \\ \\ + \dfrac{M^\frac{p}{2p-1}}{\sqrt{t-t_0+T}} + \sup _{Q_{R, T}}\left\{ v^{\frac{p}{2p-1}} [\Sigma ^\star _v(t,x,v)]_+^{1/2}\right\} \end{array} \right\} . \end{aligned}$$

### Remark 7.2

Since *u* and *v* are related to one-another via $u=v^{1/(p-1/2)}$ we can write (7.1) as $\Sigma ^\star (t,x,v) = (p-1/2) u^{(2p-3)/2} {{\mathscr {N}}}(t,x,u)$.

Subject to global bounds we have the following global estimate (in space) that results from passing to the limit $R \rightarrow \infty$ in (7.2).

### Theorem 7.3

Let $(M, g, d\mu )$ be a complete smooth metric measure space with $d\mu =e^{-f} dv_g$. Suppose that the metric and potential are time dependent, of class $\mathscr {C}^2$ and that for suitable constants $k, h \ge 0$ and $m \ge n$ satisfy ${{\mathscr {R}}ic}_f^m (g) \ge -(m-1)kg$, $\partial _t g \ge -2\,h g$ on $M \times [t_0-T, t_0]$. Let *u* be a positive solution to (1.1) with $p_0< p < 1$ and let $v = u^{p-1/2}$ and $M=\sup v$. Then there exists $C=C(p,m)>0$ such that for every $x \in M$ and $t_0-T<t <t_0$ we have

7.3 $$\begin{aligned} |\nabla v|(x,t) \le C \left\{ \begin{array}{ll} \left[ \sqrt{h} + \dfrac{1}{\sqrt{t-t_0+T}} \right] M^\frac{p}{2p-1} + \sqrt{k} M \\ \\ + \sup _{M \times [t_0-T, t_0]}\left\{ v^{\frac{p}{2p-1}} [\Sigma ^\star _v(t,x,v)]_+^{1/2}\right\} \\ \\ +\sup _{M \times [t_0-T, t_0]} \left\{ v^{\frac{2p}{3(2p-1)}} |\Sigma ^\star _x(t,x,v)|^{1/3}\right\} \end{array} \right\} \end{aligned}$$

In the static case $\partial _t g \equiv 0$ and $\partial _t f \equiv 0$ by taking $h=0$ we obtain the following local version of the estimate. The global version follows by passing to the limit $R\rightarrow \infty$.

### Theorem 7.4

Let $(M, g, d\mu )$ be a complete smooth metric measure space with $d\mu =e^{-f} dv_g$ and ${{\mathscr {R}}ic}_f^m (g) \ge -(m-1)kg$ in ${{\mathscr {B}}}_R$ for some $k \ge 0$, $m \ge n$ and $R>0$. Let *u* be a positive solution to (1.1) with $p_0< p < 1$, $v = u^{p-1/2}$ and $M= \sup _{Q_{R,T}} v$. Then there exists $C=C(p,m)>0$ such that for (*x*, *t*) in $Q_{R/2,T}$ with $t>t_0-T$ we have

7.4 $$\begin{aligned} |\nabla v|(x,t) \le C \left\{ \begin{array}{ll} \dfrac{M^\frac{p}{2p-1}}{\sqrt{t-t_0+T}} +\sup _{Q_{R, T}} \left\{ v^{\frac{2p}{3(2p-1)}} |\Sigma ^\star _x(t,x,v)|^{1/3}\right\} \\ \\ + \left[ \dfrac{k^{1/4}}{\sqrt{R}} + \dfrac{1}{R} + \sqrt{k} \right] M + \sup _{Q_{R, T}}\left\{ v^{\frac{p}{2p-1}} [\Sigma ^\star _v(t,x,v)]_+^{1/2}\right\} \end{array} \right\} . \end{aligned}$$

We end this section with an application of the estimates above to parabolic Liouville-type theorems. Compare with Theorem 2.5.

### Theorem 7.5

Let $(M, g, d\mu )$ be a complete smooth metric measure space with $d\mu =e^{-f}dv_g$ and ${{\mathscr {R}}ic}^m_f(g) \ge 0$. Assume $(3-2p) {{\mathscr {N}}}(u)/u - 2 {{\mathscr {N}}}_u(u) \ge 0$ for all $u>0$ where $p_0<p<1$. Then any positive ancient solution to the nonlinear fast diffusion equation

7.5 $$\begin{aligned} \square _p u = \partial _t u - \Delta _f u^p = {{\mathscr {N}}}(u(x,t)), \end{aligned}$$

satisfying the growth at infinity $u(x,t) = o([\varrho (x) + \sqrt{|t|}]^{2/p})$ must be spatially constant. If, in addition, ${{\mathscr {N}}}(u) \ge a$ for some $a>0$ and all $u>0$ then (7.5) admits no such ancient solutions.

## Evolution inequalities $\textbf{II}$: ${{\mathscr {L}}} = \partial _t- pv^{2(p-1)/(2p-1)} \Delta _f$ acting on $w = |\nabla v|^2$

In this section we derive evolution inequalities for $w = |\nabla v|^2$ where *v* relates to *u* through $v=u^{p-1/2}$ and ${\mathscr {L}} = \partial _t- pv^{2(p-1)/(2p-1)} \Delta _f$.

### Lemma 8.1

Let *u* be a positive solution to (1.1) with $1/2<p<1$ and let $v = u^{p-1/2}$ and $\Sigma ^\star =\Sigma ^\star (t,x,v)$ be as in (7.1). Then *v* satisfies the evolution equation

8.1 $$\begin{aligned} {{\mathscr {L}}}[v] = \left[ \partial _t- pv^{2(p-1)/(2p-1)} \Delta _f \right] v = \frac{p}{2p-1} v^{\frac{1}{1-2p}} |\nabla v|^2 + \Sigma ^\star (t,x,v). \end{aligned}$$

### Proof

As here $u = v^{\frac{1}{p-1/2}}$ a straightforward calculation results in the relations

8.2 $$\begin{aligned} \partial _t u&=\partial _t v^{\frac{1}{p-1/2}} = \frac{2v^{\frac{1}{p-1/2}-1}}{2p-1} \partial _t v, \nonumber \\ \Delta _f u^p&= \frac{2pv^{\frac{p}{p-1/2}-1}}{2p-1} \left[ \Delta v + \frac{1}{2p-1} \frac{|\nabla v|^2}{v}\right] - \frac{2pv^{\frac{p}{p-1/2}-1}}{2p-1} \langle \nabla f , \nabla v \rangle . \end{aligned}$$

Using these ingredients in (1.1) then gives

$$\begin{aligned}&\square _p u = \partial _t u - \Delta _f u^p \\&\quad = \frac{2v^{\frac{1}{p-1/2}-1}}{2p-1} \partial _t v -\frac{2pv^{\frac{p}{p-1/2}-1}}{2p-1} \left[ \Delta _f v + \frac{|\nabla v |^2}{(2p-1)v} \right] = \mathscr {N}\left( t,x,v^{\frac{1}{p-1/2}}\right) \end{aligned}$$

which upon recalling (7.1) and rearranging terms leads to the desired conclusion. $\square$

### Lemma 8.2

Let $(M, g, d\mu )$ be a complete smooth metric measure space with $d\mu =e^{-f} dv_g$. Suppose that the metric and potential are time dependent and of class $\mathscr {C}^2$. Let *u* be a positive solution to (1.1) with $1/2<p<1$ and set $w = |\nabla v|^2$ where $v = u^{p-1/2}$. Then *w* satisfies the evolution inequality

8.3 $$\begin{aligned} {{\mathscr {L}}}[w] \le&~ \frac{2p[(m-1)(p-1)^2-1]}{(2p-1)^2} v^{\frac{2p}{1-2p}} w^2 +\frac{2p^2v^{\frac{1}{1-2p}}}{2p-1} \langle \nabla v , \nabla w \rangle \nonumber \\&- [\partial _t g +2p v^{\frac{p-1}{p-1/2}} {{\mathscr {R}}ic}_f^m(g)] w + 2 \langle \nabla v, \Sigma ^\star _x(t,x,v)\rangle +2 \Sigma ^\star _v(t,x,v) w. \end{aligned}$$

### Proof

Firstly, since $w = |\nabla v|^2$ and $v = u^{p-1/2}$ satisfies (8.1), a direct calculation gives

8.4 $$\begin{aligned} \partial _t w =&~ \partial _t (|\nabla v|^2) = -[\partial _t g] ( \nabla v, \nabla v) + 2 \langle \nabla v, \nabla \partial _t v \rangle \nonumber \\ =&-[\partial _t g] ( \nabla v, \nabla v) + 2\left\langle \nabla v, \nabla \left[ pv^{\frac{p-1}{p-1/2}} \Delta _f v + \frac{pv^{\frac{1}{1-2p}}}{2p-1} |\nabla v|^2 + \Sigma ^\star (t, x, v) \right] \right\rangle \nonumber \\ =&-[\partial _t g]( \nabla v, \nabla v) + \frac{4p(p -1)}{2p-1} v^{\frac{1}{1-2p}}|\nabla v|^2 \Delta _f v +2pv^{\frac{p-1}{p-1/2}} \langle \nabla v, \nabla \Delta _f v \rangle \nonumber \\&+\frac{2pv^{\frac{1}{1-2p}}}{2p-1} \langle \nabla v , \nabla |\nabla v|^2 \rangle -\frac{2pv^{\frac{2p}{1-2p}}}{(2p-1)^2} |\nabla v|^4 + 2 \langle \nabla v, \nabla \Sigma ^\star (t,x,v)\rangle . \end{aligned}$$

Next, the weighted Bochner-Weitzenböck formula as applied to *v* describes $\Delta _f w$ as

8.5 $$\begin{aligned} \Delta _f w&=\Delta _f |\nabla v|^2 =2|\nabla \nabla v|^2 +2\langle \nabla v , \nabla \Delta _f v \rangle + 2{{\mathscr {R}}ic}_f^m (\nabla v, \nabla v) + 2 \frac{\langle \nabla f , \nabla v \rangle ^2}{m-n}. \end{aligned}$$

Hence by putting (8.4)-(8.5) together, simplifying and rearranging terms it follows that

8.6 $$\begin{aligned} {[}\partial _t - pv^{\frac{p-1}{p-1/2}} \Delta _f]w =&- 2p v^{\frac{p-1}{p-1/2}} |\nabla \nabla v|^2 +\frac{4p(p -1)}{2p-1} v^{\frac{1}{1-2p}}|\nabla v|^2 \Delta _f v \nonumber \\&+\frac{2pv^{\frac{1}{1-2p}}}{2p-1} \langle \nabla v , \nabla |\nabla v|^2 \rangle -\frac{2pv^{\frac{2p}{1-2p}}}{(2p-1)^2} |\nabla v|^4\nonumber \\&- {[}\partial _t g](\nabla v, \nabla v) -2p v^{\frac{p-1}{p-1/2}} {{\mathscr {R}}ic}_f^m (\nabla v, \nabla v) \nonumber \\&-2p v^{\frac{p-1}{p-1/2}} \frac{\langle \nabla f , \nabla v \rangle ^2}{m-n} + 2 \langle \nabla v, \nabla \Sigma ^\star (t,x,v)\rangle . \end{aligned}$$

Now recalling $\Delta _f v = \Delta v - \langle \nabla f, \nabla v \rangle$, splitting the $v^{\frac{1}{1-2p}}\langle \nabla v, \nabla |\nabla v|^2 \rangle$ term and making use of the identity $\langle \nabla v, \nabla \Sigma ^\star (t,x,v) \rangle =\langle \nabla v, \Sigma ^\star _x(t,x,v) \rangle + \Sigma ^\star _v(t,x,v) |\nabla v|^2$ we have

8.7 $$\begin{aligned} {[}\partial _t-pv^{\frac{p-1}{p-1/2}} \Delta _f]w =&-2p v^{\frac{p-1}{p-1/2}} |\nabla \nabla v|^2 +\frac{4p(p -1)}{2p-1} v^{\frac{1}{1-2p}}|\nabla v|^2 [\Delta v -\langle \nabla f, \nabla v \rangle ] \nonumber \\&-\frac{4p(p-1)}{2p-1} v^{\frac{1}{1-2p}} \nabla \nabla v(\nabla v, \nabla v) +\frac{2p^2v^{\frac{1}{1-2p}}}{2p-1} \langle \nabla v , \nabla |\nabla v|^2 \rangle \nonumber \\&-\frac{2pv^{\frac{2p}{1-2p}}}{(2p-1)^2} |\nabla v|^4 -[\partial _t g + 2p v^{\frac{p-1}{p-1/2}} {{\mathscr {R}}ic}_f^m(g)] (\nabla v, \nabla v) \nonumber \\&-2p v^{\frac{p-1}{p-1/2}} \frac{\langle \nabla f , \nabla v \rangle ^2}{m-n} + 2 \langle \nabla v, \Sigma ^\star _x(t,x,v)\rangle +2 \Sigma ^\star _v(t,x,v) |\nabla v|^2. \end{aligned}$$

Setting $A=\nabla \nabla v$ with $\textrm{tr} A=\Delta v$ and $e = \nabla v/ |\nabla v|$ we can rewrite the right-hand side of (8.7) as

8.8 $$\begin{aligned} \text{ RHS } (8.7) =&- 2p v^{\frac{p-1}{p-1/2}} |A|^2 -\frac{4p(p -1)}{2p-1} v^{\frac{1}{1-2p}}\left[ \frac{A(e,e)}{|A|}-\frac{\textrm{tr} A }{|A|} \right] |A| |\nabla v|^2\nonumber \\&+\frac{2p^2v^{\frac{1}{1-2p}}}{2p-1} \langle \nabla v , \nabla |\nabla v|^2 \rangle -\frac{2pv^{\frac{2p}{1-2p}}}{(2p-1)^2} |\nabla v|^4\nonumber \\&-[\partial _t g+2p v^{\frac{p-1}{p-1/2}} {{\mathscr {R}}ic}_f^m(g)] (\nabla v, \nabla v)\nonumber \\&-2p v^{\frac{p-1}{p-1/2}} \left[ \frac{\langle \nabla f , \nabla v \rangle ^2}{m-n} +\frac{2(p -1)}{2p-1} |\nabla v|^2 \frac{\langle \nabla f, \nabla v \rangle }{v}\right] \nonumber \\&+ 2 \langle \nabla v, \Sigma ^\star _x(t,x,v)\rangle +2 \Sigma ^\star _v(t,x,v) |\nabla v|^2. \end{aligned}$$

From (8.8) upon completing squares it follows that

8.9 $$\begin{aligned} {[}\partial _t&- pv^{\frac{p-1}{p-1/2}} \Delta _f]w = -2p v^{\frac{p-1}{p-1/2}} \left[ |A| + \frac{p-1}{2p -1} \frac{|\nabla v|^2}{v} \left( \frac{A(e,e)}{|A|} - \frac{\textrm{tr} A }{|A|} \right) \right] ^2 \nonumber \\&+ \frac{2p(p-1)^2}{(2p -1)^2} v^{\frac{2p}{1-2p}} |\nabla v|^4 \left[ \frac{A(e,e)}{|A|} - \frac{\textrm{tr} A }{|A|} \right] ^2\nonumber \\&+\frac{2p^2v^{\frac{1}{1-2p}}}{2p-1} \langle \nabla v , \nabla |\nabla v|^2 \rangle -\frac{2pv^{\frac{2p}{1-2p}}}{(2p-1)^2} |\nabla v|^4\nonumber \\&-[\partial _t g+2p v^{\frac{p-1}{p-1/2}} {{\mathscr {R}}ic}_f^m(g)] (\nabla v, \nabla v) + \frac{2p(p-1)^2(m-n)}{(2p-1)^2} v^{\frac{2p}{1-2p}} |\nabla v|^4\nonumber \\&-2pv^{\frac{p-1}{p-1/2}}\left[ \frac{\langle \nabla f , \nabla v \rangle }{\sqrt{m-n}} + \frac{2(p-1)}{2p-1}|\nabla v|^2 \frac{\sqrt{m-n}}{2v}\right] ^2 \nonumber \\&+ 2 \langle \nabla v, \Sigma ^\star _x(t,x,v)\rangle +2 \Sigma ^\star _v(t,x,v) |\nabla v|^2. \end{aligned}$$

Subsequently basic considerations lead to the inequality

8.10 $$\begin{aligned} {[}\partial _t - pv^{\frac{p-1}{p-1/2}} \Delta _f]w \le&~\frac{2p(p-1)^2}{(2p -1)^2} v^{\frac{2p}{1-2p}} |\nabla v|^4 \left[ \frac{A(e,e)}{|A|} - \frac{\textrm{tr} A }{|A|} \right] ^2\nonumber \\&+\frac{2p^2v^{\frac{1}{1-2p}}}{2p-1} \langle \nabla v , \nabla |\nabla v|^2 \rangle -\frac{2pv^{\frac{2p}{1-2p}}}{(2p-1)^2} |\nabla v|^4\nonumber \\&-[\partial _t g+2p v^{\frac{p-1}{p-1/2}} {{\mathscr {R}}ic}_f^m(g)] (\nabla v, \nabla v)\nonumber \\&+\frac{2p(p-1)^2}{(2p-1)^2} (m-n) v^{\frac{2p}{1-2p}} |\nabla v|^4\nonumber \\&+ 2 \langle \nabla v, \Sigma ^\star _x(t,x,v)\rangle +2 \Sigma ^\star _v(t,x,v) |\nabla v|^2. \end{aligned}$$

We now apply the matrix variational inequality (*see*, e.g., [42] Lemma A1, pp. 1419)

8.11 $$\begin{aligned} \left[ \frac{A(e,e)}{|A|} - \frac{\textrm{tr} A}{|A|} \right] ^2 \le \max _{\begin{array}{c} A \in \textbf{Sym}_n \\ {|e|=1} \end{array}} \left[ \frac{A(e,e)}{|A|} - \frac{\textrm{tr} A}{|A|} \right] ^2 = (n-1), \end{aligned}$$

to maximise the expression on the first line on the right-hand side. After rearranging terms this gives

8.12 $$\begin{aligned} {[}\partial _t - pv^{\frac{p-1}{p-1/2}} \Delta _f]w \le&~ \frac{2p(n-1)(p-1)^2}{(2p -1)^2} v^{\frac{2p}{1-2p}} |\nabla v|^4 +\frac{2p^2v^{\frac{1}{1-2p}}}{2p-1} \langle \nabla v , \nabla |\nabla v|^2 \rangle \nonumber \\&-\frac{2pv^{\frac{2p}{1-2p}}}{(2p-1)^2} |\nabla v|^4 -[\partial _t g+2p v^{\frac{p-1}{p-1/2}} {{\mathscr {R}}ic}_f^m(g)] (\nabla v, \nabla v)\nonumber \\&+ \frac{2p(p-1)^2}{(2p-1)^2} (m-n) v^{\frac{2p}{1-2p}} |\nabla v|^4 + 2 \langle \nabla v, \Sigma ^\star _x(t,x,v)\rangle \nonumber \\&+2 \Sigma ^\star _v(t,x,v) |\nabla v|^2. \end{aligned}$$

Reformulating based on the substitution $w=|\nabla v|^2$ and rearranging terms leads at once to the desired conclusion. $\square$

## Localisation in space-time cylinders $Q_{R,T}$ and proof of Theorem 7.1

Having derived the necessary evolution inequalities, we now proceed to finalising the proof of the local estimate in Theorem 7.1. The idea is to start by considering the localised function $\eta w$ where $\eta$ is the cylindrical cut-off function in (4.2) and then relate the evolutions of $\eta w$ and *w* under ${\mathscr {L}}$ by using an identity similar to (4.4). Indeed it is easily seen that here for the evolution operator ${\mathscr {L}} =\partial _t - pv^{(p-1)/(p-1/2)} \Delta _f$ we have

9.1 $$\begin{aligned} {{\mathscr {L}}}[\eta w] = w{{\mathscr {L}}}[\eta ] - 2pv^{\frac{p-1}{p-1/2}} [\langle \nabla \eta ,\nabla (\eta w) \rangle - |\nabla \eta |^2 w]/\eta +\eta {{\mathscr {L}}}[w]. \end{aligned}$$

Using the bound on ${{\mathscr {L}}}[w]$ from (8.3) in Lemma 8.2 and the relation $\eta \langle \nabla v, \nabla w \rangle = \langle \nabla v, \nabla (\eta w) \rangle - w \langle \nabla v, \nabla \eta \rangle$ we can then deduce that

9.2 $$\begin{aligned} {{\mathscr {L}}}[\eta w] \le&~ w {{\mathscr {L}}}[\eta ] - 2pv^{\frac{p-1}{p-1/2}} \left\langle \frac{\nabla \eta }{\eta } , \nabla (\eta w) \right\rangle + 2pv^{\frac{p-1}{p-1/2}} \frac{|\nabla \eta |^2}{\eta } w \nonumber \\&+\frac{2p[(m-1)(p-1)^2-1]}{(2p-1)^2} v^{\frac{2p}{1-2p}} \eta w^2 +\frac{2p^2v^{\frac{1}{1-2p}}}{2p-1} \langle \nabla v , \nabla (\eta w) \rangle \nonumber \\&-\frac{2p^2v^{\frac{1}{1-2p}}}{2p-1} w \langle \nabla v , \nabla \eta \rangle +2 [p v^{\frac{p-1}{p-1/2}} (m-1) k+h] \eta w\nonumber \\&+ 2 \eta \langle \nabla v, \Sigma ^\star _x(t,x,v)\rangle +2 \Sigma ^\star _v(t,x,v) \eta w. \end{aligned}$$

Assume now that the localised function $\eta w$ achieves its maximum over the compact set $\{(x,t)|d(x,x_0, t) \le R, t_0-T \le t \le \tau \} \subset M \times [t_0-T,t_0]$ at the point $(x_1,t_1)$. We assume that $(\eta w)(x_1, t_1) >0$ as otherwise the conclusion of the theorem is true with $w(x, \tau ) \le 0$ for all $d(x, x_0, \tau ) \le R/2$. In particular it follows from this that $t_1>t_0-T$ and subsequently at the point $(x_1,t_1)$ we have

- $$\partial _t (\eta w) \ge 0$$,
- $$\nabla (\eta w) =0$$,
- $$\Delta _f(\eta w) \le 0$$.

As a result from the above it also follows that at the point $(x_1,t_1)$ we have

9.3 $$\begin{aligned} {\mathscr {L}} [\eta w] = [\partial _t - pv^{\frac{p-1}{p-1/2}} \Delta _f] (\eta w) \ge 0. \end{aligned}$$

Using this inequality we can rewrite (9.2) after a rearrangement of terms as

9.4 $$\begin{aligned} \frac{2p[1-(m-1)(p-1)^2]}{(2p-1)^2} v^{\frac{2p}{1-2p}} \eta w^2 \le&~ w {{\mathscr {L}}}[\eta ] +2 [p v^{\frac{p-1}{p-1/2}} (m-1) k+h] \eta w\nonumber \\&-\frac{2p^2v^{\frac{1}{1-2p}}}{2p-1} w \langle \nabla v , \nabla \eta \rangle + 2pv^{\frac{p-1}{p-1/2}} \frac{|\nabla \eta |^2}{\eta } w\nonumber \\&+ 2 \eta \langle \nabla v, \Sigma ^\star _x(t,x,v)\rangle +2 \Sigma ^\star _v(t,x,v) \eta w. \end{aligned}$$

Denoting $p[1-(m-1)(p-1)^2]/(2p-1)^2 = 1/\gamma >0$ (the positivity being a direct consequence of the imposed range of *p*) and multiplying through by $\gamma v^{2p/(2p-1)}$ gives

9.5 $$\begin{aligned} 2\eta w^2 \le&~ \gamma v^{\frac{2p}{2p-1}} w {{\mathscr {L}}}[\eta ] +2 \gamma [p v^{\frac{p-1}{p-1/2}} (m-1) k+h] v^{\frac{2p}{2p-1}} \eta w\nonumber \\&-\frac{2 \gamma p^2}{2p-1} v w \langle \nabla v , \nabla \eta \rangle + 2 \gamma p v^2 \frac{|\nabla \eta |^2}{\eta } w\nonumber \\&+ 2 \gamma \eta v^{\frac{2p}{2p-1}} \langle \nabla v, \Sigma ^\star _x(t,x,v)\rangle +2 \gamma v^{\frac{2p}{2p-1}}\Sigma ^\star _v(t,x,v) \eta w. \end{aligned}$$

Now we proceed onto bounding the expression on the right-hand side of (9.5). As this is similar to the proof of Theorem 2.1 we shall remain brief, focusing mainly on the differences. Starting from the first term and arguing as in Theorem 2.1 we have

9.6 $$\begin{aligned} \gamma v^{\frac{2p}{2p-1}} w {\mathscr {L}}[\eta ] =&~ \gamma v^{\frac{2p}{2p-1}} w[\partial _t - pv^{\frac{p-1}{p-1/2}} \Delta _f] \eta = \gamma v^{\frac{2p}{2p-1}} w \partial _t \eta - \gamma p v^2 w \Delta _f \eta \nonumber \\ \le&~ \frac{1}{6} \eta w^2 + C \gamma ^2 \left[ \frac{1}{(\tau -t_0+T)^2} + h^2\right] (\sup _{Q_{R, T}} v)^{\frac{4p}{2p-1}}\nonumber \\&+ C \gamma ^2p^2 (m-1)^2\left[ \frac{1+ k R^2}{R^4}\right] (\sup _{Q_{R, T}} v)^{4}. \end{aligned}$$

Next, for the second and third terms, we can write

9.7 $$\begin{aligned}&2 \gamma [p v^{\frac{p-1}{p-1/2}} (m-1) k] v^{\frac{2p}{2p-1}} \eta w \nonumber \\&= 2 \gamma p (m-1) k v^2 \eta w \le \frac{1}{6} \eta w^2 + C \gamma ^2 p^2 (m-1)^2 k^2(\sup _{Q_{R, T}} v)^4, \nonumber \\ 2 \gamma h v^{\frac{2p}{2p-1}} \eta w&\le 2 \gamma h v^{\frac{2p}{2p-1}} \eta ^{1/2} w \le \frac{1}{6} \eta w^2 + C \gamma ^2 h^2 (\sup _{Q_{R, T}} v)^{\frac{4p}{2p-1}}. \end{aligned}$$

We can bound the fourth term by writing

9.8 $$\begin{aligned} -\frac{2 \gamma p^2}{2p-1} v w \langle \nabla v , \nabla \eta \rangle \le \frac{2 \gamma p^2}{2p-1} vw |\nabla v| |\nabla \eta |&\le \frac{2 \gamma p^2}{2p-1} v \eta ^{3/4} w^{3/2} \frac{|\nabla \eta |}{\eta ^{3/4}}\nonumber \\ \le \frac{1}{6} (\eta ^{3/4} w^{3/2})^{4/3} + \frac{C\gamma ^4 p^8}{(2p-1)^4} \left[ \frac{|\nabla \eta |}{\eta ^{3/4}}\right] ^4 v^4&\le \frac{1}{6} \eta w^2 + \frac{C \gamma ^4 p^8}{(2p-1)^4 R^4} (\sup _{Q_{R, T}} v)^4. \end{aligned}$$

In much the same way

9.9 $$\begin{aligned} 2 \gamma p v^2 \frac{|\nabla \eta |^2}{\eta } w \le \frac{1}{6} \eta w^2 + \frac{C \gamma ^2 p^2}{R^4}(\sup _{Q_{R, T}} v)^4. \end{aligned}$$

Finally for the $\Sigma ^\star (t,x,v)$ terms, upon recalling $0\le \eta \le 1$ and utilising Young’s inequality we have

9.10 $$\begin{aligned} 2 \gamma \eta w v^{\frac{2p}{2p-1}}\Sigma ^\star _v(t,x,v) \le \frac{1}{6} \eta w^2 + C \gamma ^2 \sup _{Q_{R, T}} \left\{ v^{\frac{4p}{2p-1}} \left[ \Sigma ^\star _v(t,x,v) \right] _+^2\right\} . \end{aligned}$$

Furthermore, by using Cauchy-Schwarz and Young inequalities we can write

9.11 $$\begin{aligned} 2 \gamma \eta v^{\frac{2p}{2p-1}} \langle \nabla v, \Sigma ^\star _x(t,x,v) \rangle \le&~ 2 \gamma \eta v^{\frac{2p}{2p-1}} |\nabla v| |\Sigma ^\star _x(t,x,v)| \nonumber \\ \le&~ \frac{1}{6} \eta w^2+C \gamma ^{4/3} \sup _{Q_{R, T}} \left\{ v^{\frac{8p}{3(2p-1)}} |\Sigma ^\star _x(t,x,v)|^{4/3}\right\} . \end{aligned}$$

Completing the estimate of the individual terms on the right-hand side of (9.5), by inserting these estimates back and after basic considerations and adjusting the constants at the space-time point $(x_1, t_1)$, we arrive at the following bound:

9.12 $$\begin{aligned} \eta w^2 \le&~C\gamma ^2 \left\{ \begin{array}{ll} p^2 (m-1)^2\left[ \dfrac{1+ k R^2}{R^4}\right] +\dfrac{p^2}{R^4} \\ \\ +\dfrac{\gamma ^2 p^8}{(2p-1)^4 R^4} +p^2 (m-1)^2 k^2 \end{array} \right\} (\sup _{Q_{R, T}} v)^4 \nonumber \\&+C \left\{ \begin{array}{ll} \gamma ^{2} \left[ \dfrac{1}{(\tau -t_0+T)^2} + 2h^2\right] (\sup _{Q_{R, T}} v)^{\frac{4p}{2p-1}} \\ \\ +\gamma ^{4/3}\sup _{Q_{R, T}} \left\{ v^{\frac{8p}{3(2p-1)}} |\Sigma ^\star _x(t,x,v)|^{4/3}\right\} \\ \\ + \gamma ^{2} \sup _{Q_{R, T}} \left\{ v^{\frac{4p}{2p-1}} \left[ \Sigma ^\star _v(t,x,v) \right] _+^2\right\} \end{array} \right\} . \end{aligned}$$

Upon adjusting terms, absorbing $\gamma$ into *C* and recalling $M=\sup _{Q_{R,T}} v$ it follows that

9.13 $$\begin{aligned} \eta w^2 \le&~C(\gamma ) \left\{ \left( \begin{array}{ll} p^2 (m-1)^2\left[ \dfrac{1+ k R^2}{R^4}\right] +\dfrac{p^2}{R^4} \\ \\ +\dfrac{p^8}{(2p-1)^4 R^4} + p^2 (m-1)^2 k^2 \end{array} \right) \right. M^4 \nonumber \\&\qquad \qquad + \left. \left( \begin{array}{ll} \left[ \dfrac{1}{(\tau -t_0+T)^2} + 2h^2 \right] M^\frac{4p}{2p-1} \\ \\ + \sup _{Q_{R, T}} \left\{ v^{\frac{8p}{3(2p-1)}} |\Sigma ^\star _x(t,x,v)|^{4/3}\right\} \\ \\ +\sup _{Q_{R, T}} \left\{ v^{\frac{4p}{2p-1}} [\Sigma ^\star _v(t,x,v)]_+^2\right\} \end{array} \right) \right\} . \end{aligned}$$

Recalling the maximality of $\eta w$ at $(x_1, t_1)$ along with $\eta \equiv 1$ when $d(x, x_0,t) \le R/2$ and $\tau \le t \le t_0$, it follows that $w^2 (x, \tau ) = (\eta ^2 w^2)(x, \tau ) \le (\eta ^2 w^2)(x_1,t_1) \le (\eta w^2)(x_1,t_1)$. Hence noting $w = |\nabla v|^2$ where $v = u^{p-1/2}$ and absorbing *p* and *m* into $C(\gamma )$ gives

9.14 $$\begin{aligned} |\nabla v|(x,\tau ) \le C \left\{ \begin{array}{ll} \left[ \dfrac{k^{1/4}}{\sqrt{R}} +\dfrac{1}{R} +\sqrt{k} \right] M +\sqrt{h} M^\frac{p}{2p-1} \\ \\ + \dfrac{M^\frac{p}{2p-1}}{\sqrt{\tau -t_0+T}} + \sup _{Q_{R, T}}\left\{ v^{\frac{p}{2p-1}} [\Sigma ^\star _v(t,x,v)]_+^{1/2}\right\} \\ \\ + \sup _{Q_{R, T}} \left\{ v^{\frac{2p}{3(2p-1)}} |\Sigma ^\star _x(t,x,v)|^{1/3}\right\} \end{array} \right\} , \end{aligned}$$

where $C=C(p,m)>0$. Making note of the arbitrariness of $\tau$ in the interval $(t_0-T, t_0]$ gives the desired conclusion. $\square$

## Ancient solutions to $$\partial _t u - \Delta _f u^p = {{\mathscr {N}}}(u)$$$$\textbf{II}$$: proof of Theorem 7.5

Let us now present the proof of Theorem 7.5. We first fix a space-time point $(x_0,t_0)$. Then with the choices $t=t_0$, $R>0$ and $T=R^2$ it follows from (7.4) in Theorem 7.4 (with $k=0$) that

10.1 $$\begin{aligned} |\nabla v|(x_0,t_0)&\le C \left\{ \begin{array}{ll} \dfrac{M^\frac{p}{2p-1}}{\sqrt{t-t_0+T}} +\sup _{Q_{R, T}} \left\{ v^{\frac{2p}{3(2p-1)}} |\Sigma ^\star _x(t,x,v)|^{1/3}\right\} \\ \\ +\left[ \dfrac{k^{1/4}}{\sqrt{R}} + \dfrac{1}{R} + \sqrt{k} \right] M + \sup _{Q_{R, T}}\left\{ v^{\frac{p}{2p-1}} [\Sigma ^\star _v(t,x,v)]_+^{1/2}\right\} \end{array} \right\} \nonumber \\&\le C \left\{ \begin{array}{ll} \dfrac{M^\frac{p}{2p-1}}{\sqrt{T}} +\sup _{Q_{R, T}} \left\{ v^{\frac{2p}{3(2p-1)}} |\Sigma ^\star _x(t,x,v)|^{1/3} \right\} \\ \\ + \dfrac{M}{R} + \sup _{Q_{R, T}} \left\{ v^{\frac{p}{2p-1}}\left[ \Sigma ^\star _v (t,x,v)\right] _+^{1/2} \right\} \end{array} \right\} . \end{aligned}$$

Now $\Sigma ^\star (t,x,v) = (p-1/2) u^{(2p-3)/2} {{\mathscr {N}}}(u)$ where $u=v^{1/(p-1/2)}$ (*see* (7.5) and Remark 7.2). Hence it is seen that $\Sigma ^\star$ does not depend explicitly on *t* and *x* and so $\Sigma ^\star = \Sigma ^\star (v)$. In particular $\Sigma ^\star _x \equiv 0$ and by a direct calculation using $\Sigma ^\star _v (v) = \Sigma ^\star _u (v) \partial _v u$ that

10.2 $$\begin{aligned} \Sigma ^\star _v (v)&= (p-1/2) [(p-3/2) u^{p-5/2} {{\mathscr {N}}} (u)+ u^{p-3/2} {{\mathscr {N}}}_u(u)] u^{(3-2p)/2} /(p-1/2) \nonumber \\&= (p-3/2) {{\mathscr {N}}}(u)/u + {{\mathscr {N}}}_u(u). \end{aligned}$$

From the assumptions on ${{\mathscr {N}}}$ it now follows that $\Sigma ^\star _v (v) \le 0$ and as a result $[\Sigma ^\star _v]_+ \equiv 0$. Next from the growth assumption on *u* we have

10.3 $$\begin{aligned} v = u^{p-1/2} = \left[ o([\varrho (x) + \sqrt{|t|}]^{2/p}) \right] ^{p-1/2} = o([\varrho (x) + \sqrt{|t|}]^{(2p-1)/p}), \end{aligned}$$

and so as a result $M=\sup _{Q_{R,T}} v =o(R^{(2p-1)/p})$. Returning now to (10.1), using the above we have

10.4 $$\begin{aligned} |\nabla v| (x_0,t_0) \le C \left[ \frac{M^\frac{p}{2p-1}}{\sqrt{T}}+\frac{M}{R} \right] \le \frac{o(R)}{R} + \frac{o(R^{(2p-1)/p})}{R}. \end{aligned}$$

Passing to the limit $R \nearrow \infty$ and noting that $(2p-1)/p \le 1$ in the range $1/2<p<1$ it follows that $|\nabla v|(x_0,t_0)=0$. The arbitrariness of $(x_0,t_0)$ implies $|\nabla v| \equiv 0$ and so *v* and subsequently *u* are spatially constant. Hence we have $u=u(t)$. From the equation satisfied by *u* it then follows that $du/dt = {{\mathscr {N}}}(u)$. The rest of the proof proceeds as in the proof of Theorem 2.5. $\square$

## The Ricci-Perelman super flow $$\textbf{II}$$: $$\partial _t g + 2p v^{2(p-1)/(2p-1)}$$$${{\mathscr {R}}ic}^m_f(g) \ge - 2\textsf{k}g$$

In this final section of the paper we derive further global gradient bounds on positive smooth solutions to equation (1.1). Here *M* is closed and the metric and potential are assumed to evolve under the super flow [*cf.* (1.11) and (1.13)]

11.1 $$\begin{aligned} \frac{1}{2} \dfrac{\partial g}{\partial t} (x,t) + p v^{2(p-1)/(2p-1)}(x,t) {{\mathscr {R}}ic}^m_f(g)(x,t) \ge - \textsf{k}g(x,t), \qquad {{\textsf{k}}} \ge 0. \end{aligned}$$

### Lemma 11.1

Let *u* be a positive solution to (1.1) and $v = u^{p-1/2}$ where $1/2<p<1$. For $s \ge 2$ and $\zeta =\zeta (t)$ non-negative and of class ${{\mathscr {C}}}^1[0, \infty )$ let

11.2 $$\begin{aligned} {{\textsf{H}}}^{s}_\zeta [v] = \zeta (t) |\nabla v|^s + \Gamma (v), \end{aligned}$$

where $\Gamma =\Gamma (v)$ is of class $\mathscr {C}^2(0, \infty )$. Then ${{\textsf{H}}} = {{\textsf{H}}}^{s}_\zeta [v]$ satisfies the evolution identity

11.3 $$\begin{aligned} {{\mathscr {L}}} ({{\textsf{H}}}^{s}_\zeta [v]) -&\frac{2p^2v^{\frac{1}{1-2p}}}{2p-1} \langle \nabla v , \nabla {{\textsf{H}}}^{s}_\zeta [v] \rangle \nonumber \\ =&~\zeta '(t) |\nabla v|^s - s\zeta (t) |\nabla v|^{s-2} \left[ \frac{1}{2} \partial _t g + pv^{\frac{p-1}{p-1/2}} {{\mathscr {R}}ic}_f^m(g) \right] ( \nabla v, \nabla v) \nonumber \\&- \frac{2 sp(p-1)}{2p-1} \zeta (t) v^{\frac{1}{1-2p}} |\nabla v|^{s-2} \nabla \nabla v (\nabla v, \nabla v) - \frac{sp \zeta (t)}{(2p-1)^2} v^{\frac{2p}{1-2p}} |\nabla v|^{s+2} \nonumber \\&- sp\zeta (t) v^{\frac{p-1}{p-1/2}} |\nabla v|^{s-2} |\nabla \nabla v|^2 + \frac{2sp(p -1)}{2p-1} \zeta (t) v^{\frac{1}{1-2p}} |\nabla v|^s \Delta v \nonumber \\&- \frac{s}{2} \zeta (t) pv^{\frac{p-1}{p-1/2}} \langle \nabla |\nabla v|^{s-2} , \nabla |\nabla v|^2 \rangle - \frac{2sp(p -1)}{2p-1} \zeta (t) v^{\frac{1}{1-2p}} |\nabla v|^s \langle \nabla f, \nabla v \rangle \nonumber \\&- sp \zeta (t) v^{\frac{p-1}{p-1/2}} |\nabla v|^{s-2} \langle \nabla f , \nabla v \rangle ^2/(m-n) + s \zeta (t) |\nabla v|^{s-2}\langle \nabla v, \nabla \Sigma ^\star (t,x,v)\rangle \nonumber \\&+\Gamma '(v) \Sigma ^\star (t,x,v) -p v^{\frac{1}{1-2p}} [\Gamma '(v)+v\Gamma ''(v)] |\nabla v|^2. \end{aligned}$$

### Proof

Referring to (11.2) it is a straightforward matter to see that

11.4 $$\begin{aligned} {{\mathscr {L}}}({{\textsf{H}}}^{s}_\zeta [v]) = \zeta (t) {\mathscr {L}}[|\nabla v|^s ] + \zeta '(t) |\nabla v|^s + {\mathscr {L}}[\Gamma (v)]. \end{aligned}$$

We now go forward by evaluating the expressions in the first and third terms on the right-hand side. For the first term, ignoring the factor $\zeta (t)$ for the moment, we have

11.5 $$\begin{aligned} {{\mathscr {L}}} [|\nabla v|^s] =&~\frac{s}{2} |\nabla v|^{s-2} \left[ \partial _t -pv^{\frac{p-1}{p-1/2}} \Delta _f\right] |\nabla v|^2 - \frac{s}{2}pv^{\frac{p-1}{p-1/2}} \langle \nabla |\nabla v|^{s-2} , \nabla |\nabla v|^2 \rangle , \end{aligned}$$

where by a direct calculation using Lemma 8.1 and (1.5) it is seen that

11.6 $$\begin{aligned} \left[ \partial _t -pv^{\frac{p-1}{p-1/2}} \Delta _f\right] |\nabla v|^2 =&-[\partial _t g] ( \nabla v, \nabla v) +\frac{4p(p -1)}{2p-1} v^{\frac{1}{1-2p}} |\nabla v|^2 \Delta _f v \\&+\frac{2pv^{\frac{1}{1-2p}}}{2p-1} \langle \nabla v , \nabla |\nabla v|^2 \rangle -\frac{2pv^{\frac{2p}{1-2p}} |\nabla v|^4}{(2p-1)^2} \nonumber \\&+2 \langle \nabla v, \nabla \Sigma ^\star (t,x,v) \rangle -2 pv^{\frac{p-1}{p-1/2}} |\nabla \nabla v|^2\nonumber \\&-2 pv^{\frac{p-1}{p-1/2}} {{\mathscr {R}}ic}_f^m (\nabla v, \nabla v) - 2 pv^{\frac{p-1}{p-1/2}} \frac{\langle \nabla f , \nabla v \rangle ^2}{m-n}. \nonumber \end{aligned}$$

Therefore by combining the last two identities it follows that

11.7 $$\begin{aligned} {{\mathscr {L}}}[|\nabla v|^s] =&-\frac{s}{2} |\nabla v|^{s-2} [\partial _t g] ( \nabla v, \nabla v) + \frac{2sp(p -1)}{2p-1} v^{\frac{1}{1-2p}} |\nabla v|^s \Delta _f v\nonumber \\&+ \frac{spv^{\frac{1}{1-2p}}}{2p-1} |\nabla v|^{s-2} \langle \nabla v, \nabla |\nabla v|^2 \rangle - \frac{spv^{\frac{2p}{1-2p}}}{(2p-1)^2} |\nabla v|^{s+2}\nonumber \\&+ s |\nabla v|^{s-2}\langle \nabla v, \nabla \Sigma ^\star (t,x,v) \rangle - s pv^{\frac{p-1}{p-1/2}} |\nabla v|^{s-2} |\nabla \nabla v|^2\nonumber \\&- s pv^{\frac{p-1}{p-1/2}} |\nabla v|^{s-2} {{\mathscr {R}}ic}_f^m (\nabla v, \nabla v) -s pv^{\frac{p-1}{p-1/2}} |\nabla v|^{s-2} \frac{\langle \nabla f , \nabla v \rangle ^2}{m-n}\nonumber \\&-\frac{s}{2} pv^{\frac{p-1}{p-1/2}} \langle \nabla |\nabla v|^{s-2} , \nabla |\nabla v|^2 \rangle . \end{aligned}$$

In a similar way for the third term on the right-hand side of (11.4) we can write

11.8 $$\begin{aligned} {{\mathscr {L}}}[\Gamma (v)]&= \Gamma '(v) \left[ \frac{pv^{\frac{1}{1-2p}} |\nabla v|^2}{2p-1} + \Sigma ^\star (t, x, v)\right] - pv^{\frac{p-1}{p-1/2}} \Gamma ''(v) |\nabla v|^2, \end{aligned}$$

where we have used (8.1). Substituting (11.7) and (11.8) back into (11.4) now leads to

11.9 $$\begin{aligned} {{\mathscr {L}}} ({{\textsf{H}}}^{s}_\zeta [v]) =&~ \zeta '(t) |\nabla v|^s - s\zeta (t) |\nabla v|^{s-2}\left[ \frac{1}{2}\partial _t g + pv^{\frac{p-1}{p-1/2}} {{\mathscr {R}}ic}_f^m(g)\right] ( \nabla v, \nabla v) \nonumber \\&+ \frac{2sp(p -1)}{2p-1} \zeta (t) v^{\frac{1}{1-2p}} |\nabla v|^s [\Delta v - \langle \nabla f, \nabla v \rangle ] - sp \zeta (t)v^{\frac{p-1}{p-1/2}} |\nabla v|^{s-2} |\nabla \nabla v|^2\nonumber \\&+ \frac{sp \zeta (t)}{2p-1} v^{\frac{1}{1-2p}} |\nabla v|^{s-2}\langle \nabla v, \nabla |\nabla v|^2 \rangle -\frac{sp \zeta (t)}{(2p-1)^2} v^{\frac{2p}{1-2p}} |\nabla v|^{s+2} \nonumber \\&- sp\zeta (t)v^{\frac{p-1}{p-1/2}} |\nabla v|^{s-2} \frac{\langle \nabla f, \nabla v \rangle ^2}{m-n} - \frac{sp}{2} \zeta (t) v^{\frac{p-1}{p-1/2}} \langle \nabla |\nabla v|^{s-2} , \nabla |\nabla v|^2 \rangle \nonumber \\&+ s\zeta (t)|\nabla v|^{s-2}\langle \nabla v, \nabla \Sigma ^\star (t,x,v) \rangle - pv^{\frac{p-1}{p-1/2}} \Gamma ''(v) |\nabla v|^2\nonumber \\&+ \Gamma '(v) \left[ \frac{p v^{\frac{1}{1-2p}} |\nabla v|^2}{2p-1} + \Sigma ^\star (t, x, v)\right] . \end{aligned}$$

Next, upon rearranging terms and splitting the term $v^{\frac{1}{1-2p}} |\nabla v|^{s-2}\langle \nabla v, \nabla |\nabla v|^2 \rangle$ we have

11.10 $$\begin{aligned} {{\mathscr {L}}}({{\textsf{H}}}^{s}_\zeta [v]) =&- sp\zeta (t) v^{\frac{p-1}{p-1/2}} |\nabla v|^{s-2} |\nabla \nabla v|^2 + \frac{2sp(p -1)}{2p-1} \zeta (t) v^{\frac{1}{1-2p}} |\nabla v|^s \Delta v \nonumber \\&-\frac{2 sp(p-1)}{2p-1} \zeta (t) v^{\frac{1}{1-2p}} |\nabla v|^{s-2} \nabla \nabla v (\nabla v, \nabla v) + \zeta '(t) |\nabla v|^s \nonumber \\&+\frac{sp^2 \zeta (t)}{2p-1} v^{\frac{1}{1-2p}} |\nabla v|^{s-2} \langle \nabla v , \nabla |\nabla v|^2 \rangle -\frac{sp\zeta (t)}{(2p-1)^2} v^{\frac{2p}{1-2p}} |\nabla v|^{s+2} \nonumber \\&- s\zeta (t) |\nabla v|^{s-2}\left[ \frac{1}{2}\partial _t g + pv^{\frac{p-1}{p-1/2}} {{\mathscr {R}}ic}_f^m(g)\right] (\nabla v, \nabla v) \nonumber \\&- \frac{sp}{2} \zeta (t) v^{\frac{p-1}{p-1/2}} \langle \nabla |\nabla v|^{s-2} , \nabla |\nabla v|^2 \rangle - \frac{2sp(p -1)}{2p-1} \zeta (t) v^{\frac{1}{1-2p}} |\nabla v|^s \langle \nabla f, \nabla v \rangle \nonumber \\&- sp\zeta (t)v^{\frac{p-1}{p-1/2}} |\nabla v|^{s-2} \frac{\langle \nabla f, \nabla v \rangle ^2}{m-n} + s\zeta (t)|\nabla v|^{s-2}\langle \nabla v, \nabla \Sigma ^\star (t,x,v) \rangle \nonumber \\&- pv^{\frac{p-1}{p-1/2}} \Gamma ''(v) |\nabla v|^2 + \Gamma '(v) \left[ \frac{pv^{\frac{1}{1-2p}} |\nabla v|^2}{2p-1} + \Sigma ^\star (t, x, v)\right] . \end{aligned}$$

Taking advantage of the relation $2\langle \nabla v, \nabla |\nabla v|^s \rangle = s |\nabla v|^{s-2} \langle \nabla v, |\nabla v|^2 \rangle$ then gives

11.11 $$\begin{aligned} {{\mathscr {L}}}({{\textsf{H}}}^{s}_\zeta [v]) -&\frac{2p^2v^{\frac{1}{1-2p}}}{2p-1} \langle \nabla v , \nabla {{\textsf{H}}}^{s}_\zeta [v] \rangle \nonumber \\ =&- sp\zeta (t)v^{\frac{p-1}{p-1/2}} |\nabla v|^{s-2} |\nabla \nabla v|^2 + \frac{2sp(p -1)}{2p-1} \zeta (t)v^{\frac{1}{1-2p}} |\nabla v|^s \Delta v \nonumber \\&-\frac{2 sp(p-1)}{2p-1} \zeta (t)v^{\frac{1}{1-2p}} |\nabla v|^{s-2} \nabla \nabla v (\nabla v, \nabla v) -\frac{sp \zeta (t)}{(2p-1)^2} v^{\frac{2p}{1-2p}} |\nabla v|^{s+2} \nonumber \\&+ \zeta '(t) |\nabla v|^s - s\zeta (t)|\nabla v|^{s-2}\left[ \frac{1}{2}\partial _t g + pv^{\frac{p-1}{p-1/2}} {{\mathscr {R}}ic}_f^m(g)\right] (\nabla v, \nabla v) \nonumber \\&- \frac{sp}{2} \zeta (t) v^{\frac{p-1}{p-1/2}} \langle \nabla |\nabla v|^{s-2} , \nabla |\nabla v|^2 \rangle - s\zeta (t) \frac{2p(p -1)}{2p-1} v^{\frac{1}{1-2p}} |\nabla v|^s \langle \nabla f, \nabla v \rangle \nonumber \\&- sp\zeta (t)v^{\frac{p-1}{p-1/2}} |\nabla v|^{s-2} \frac{\langle \nabla f, \nabla v \rangle ^2}{m-n} + s\zeta (t) |\nabla v|^{s-2}\langle \nabla v, \nabla \Sigma ^\star (t,x,v) \rangle \nonumber \\&+ \Gamma '(v) \Sigma ^\star (t, x, v) - p v^{\frac{1}{1-2p}} \Gamma '(v) |\nabla v|^2 - pv^{\frac{p-1}{p-1/2}} \Gamma ''(v) |\nabla v|^2, \end{aligned}$$

which at once gives the desired conclusion. $\square$

### Lemma 11.2

Under the assumptions of Lemma 11.1, if the metric and potential evolve under the super flow (11.1), then ${{\textsf{H}}} = {{\textsf{H}}}^{s}_\zeta [v]$ satisfies the evolution inequality

11.12 $$\begin{aligned} {{\mathscr {L}}}&({{\textsf{H}}}^{s}_\zeta [v]) - \frac{2p^2v^{\frac{1}{1-2p}}}{2p-1} \langle \nabla v , \nabla {{\textsf{H}}}^{s}_\zeta [v] \rangle \nonumber \\ \le&[\zeta '(t)+ s {\textsf{k}} \zeta (t) + s \zeta (t) \Sigma _v^\star (t,x,v)] |\nabla v|^s + \frac{s p[(p-1)^2(m-1)-1]}{(2p-1)^2} \zeta (t) v^{\frac{2p}{1-2p}} |\nabla v|^{s+2} \nonumber \\&+ s \zeta (t) |\nabla v|^{s-2}\langle \nabla v, \Sigma ^\star _x(t,x,v) \rangle + \Gamma '(v) \Sigma ^\star (t,x,v) - pv^{\frac{1}{1-2p}} [\Gamma '(v)+v\Gamma ''(v)] |\nabla v|^2. \end{aligned}$$

### Proof

We start with identity (11.3) in Lemma 11.1. Using $A=\nabla \nabla v$ with $\textrm{tr} A=\Delta v$ and $e = \nabla v/ |\nabla v|$ we can rewrite this upon substitution and a rearrangement of terms in the form

11.13 $$\begin{aligned} {{\mathscr {L}}}&({{\textsf{H}}}^{s}_\zeta [v]) - \frac{2p^2v^{\frac{1}{1-2p}}}{2p-1} \langle \nabla v , \nabla {{\textsf{H}}}^{s}_\zeta [v] \rangle \nonumber \\ =&- sp\zeta (t) v^{\frac{p-1}{p-1/2}} |\nabla v|^{s-2} |A|^2 - \frac{2sp(p -1)}{2p-1} \zeta (t) v^{\frac{1}{1-2p}} \left[ \frac{A (e, e)}{|A|}- \frac{\textrm{tr} A}{|A|}\right] |\nabla v|^s |A| \nonumber \\&-\frac{sp\zeta (t)}{(2p-1)^2} v^{\frac{2p}{1-2p}} |\nabla v|^{s+2} -s\zeta (t)|\nabla v|^{s-2}\left[ \frac{1}{2}\partial _t g + pv^{\frac{p-1}{p-1/2}} {{\mathscr {R}}ic}_f^m(g)\right] ( \nabla v, \nabla v)\nonumber \\&+ \zeta '(t) |\nabla v|^s-\frac{sp}{2} \zeta (t) v^{\frac{p-1}{p-1/2}} \langle \nabla |\nabla v|^{s-2} , \nabla |\nabla v|^2 \rangle + s\zeta (t) |\nabla v|^{s-2}\langle \nabla v, \nabla \Sigma ^\star (t,x,v) \rangle \nonumber \\&- sp\zeta (t) v^{\frac{p-1}{p-1/2}} |\nabla v|^{s-2} \left[ \frac{\langle \nabla f, \nabla v \rangle ^2}{(m-n)} + \frac{2(p -1)}{2p-1} |\nabla v|^2 \frac{\langle \nabla f, \nabla v \rangle }{v}\right] \nonumber \\&+\Gamma '(v) \Sigma ^\star (t,x,v) -p v^{\frac{1}{1-2p}} [\Gamma '(v)+v\Gamma ''(v)] |\nabla v|^2. \end{aligned}$$

Upon forming a complete square for the matrix term it then follows that

11.14 $$\begin{aligned} {{\mathscr {L}}}&({{\textsf{H}}}^{s}_\zeta [v]) - \frac{2p^2 v^{\frac{1}{1-2p}}}{2p-1} \langle \nabla v , \nabla {{\textsf{H}}}^{s}_\zeta [v] \rangle \nonumber \\ =&- sp\zeta (t)v^{\frac{p-1}{p-1/2}} |\nabla v|^{s-2} \left[ |A| + \frac{p -1}{2p-1} \frac{|\nabla v|^2}{v} \left( \frac{A (e, e)}{|A|}- \frac{\textrm{tr} A}{|A|}\right) \right] ^2\nonumber \\&+ \frac{s p(p-1)^2}{(2p-1)^2} \left[ \frac{A (e, e)}{|A|}- \frac{\textrm{tr} A}{|A|}\right] ^2 \zeta (t) v^{\frac{2p}{1-2p}} |\nabla v|^{s+2} - \frac{sp \zeta (t)}{(2p-1)^2} v^{\frac{2p}{1-2p}} |\nabla v|^{s+2}\nonumber \\&+ \zeta '(t) |\nabla v|^s - s\zeta (t) |\nabla v|^{s-2}\left[ \frac{1}{2}\partial _t g + pv^{\frac{p-1}{p-1/2}} {{\mathscr {R}}ic}_f^m(g)\right] ( \nabla v, \nabla v)\nonumber \\&- \frac{sp}{2} \zeta (t) v^{\frac{p-1}{p-1/2}} \langle \nabla |\nabla v|^{s-2} , \nabla |\nabla v|^2 \rangle + \zeta (t) s |\nabla v|^{s-2}\langle \nabla v, \nabla \Sigma ^\star (t,x,v) \rangle \nonumber \\&-sp\zeta (t)v^{\frac{p-1}{p-1/2}} |\nabla v|^{s-2} \left[ \frac{\langle \nabla f, \nabla v \rangle }{\sqrt{m-n}} + \frac{2(p -1)}{2p-1} |\nabla v|^2 \frac{\sqrt{m-n}}{2v}\right] ^2 + \Gamma '(v) \Sigma ^\star (t, x, v) \nonumber \\&+\frac{sp(p-1)^2(m-n)}{(2p-1)^2} \zeta (t)v^{\frac{2p}{1-2p}} |\nabla v|^{s+2} - p v^{\frac{1}{1-2p}} [\Gamma '(v)+v\Gamma ''(v)]|\nabla v|^2. \end{aligned}$$

Now by ignoring non-positive terms and making use of the variational matrix inequality (8.11) we conclude that

11.15 $$\begin{aligned} {{\mathscr {L}}}({{\textsf{H}}}^{s}_\zeta [v]) -&\frac{2p^2v^{\frac{1}{1-2p}} }{2p-1} \langle \nabla v , \nabla {{\textsf{H}}}^{s}_\zeta [v] \rangle \le \frac{s p[(p-1)^2(m-1)-1]}{(2p-1)^2} \zeta (t) v^{\frac{2p}{1-2p}} |\nabla v|^{s+2} \nonumber \\&+ \zeta '(t) |\nabla v|^s - s\zeta (t) |\nabla v|^{s-2}\left[ \frac{1}{2}\partial _t g + pv^{\frac{p-1}{p-1/2}} {{\mathscr {R}}ic}_f^m(g)\right] ( \nabla v, \nabla v)\nonumber \\&-\frac{sp}{2} \zeta (t) v^{\frac{p-1}{p-1/2}} \langle \nabla |\nabla v|^{s-2} , \nabla |\nabla v|^2 \rangle + s\zeta (t) |\nabla v|^{s-2}\langle \nabla v, \nabla \Sigma ^\star (t,x,v) \rangle \nonumber \\&+ \Gamma '(v) \Sigma ^\star (t, x, v) - p v^{\frac{1}{1-2p}} [\Gamma '(v)+v\Gamma ''(v)]|\nabla v|^2. \end{aligned}$$

An application of inequality (11.1) along with $\langle \nabla v, \nabla \Sigma ^\star \rangle = \langle \nabla v, \Sigma ^\star _x \rangle + \Sigma ^\star _v |\nabla v|^2$ and $\langle \nabla |\nabla v|^{s-2}, \nabla |\nabla v|^2\rangle \ge 0$ when $s \ge 2$ gives the desired conclusion. $\square$

### Corollary 11.3

Let $(M,g,e^{-f} dv_g)$ be a smooth metric measure space with *M* closed. Let *u* be a positive smooth solution to (1.1) where $p_0(m)<p<1$ and set $v = u^{p-1/2}$. Assume the metric and potential satisfy the super flow (11.1) with $\textsf{k} \ge 0$. Moreover suppose the following conditions hold for some *a*:

- $$\Sigma ^\star (t,x,v) \le a$$,
- $$\Gamma '(v) \Sigma ^\star (t,x,v) \le 0$$,
- $$\Gamma '(v) + v \Gamma ''(v) \ge 0$$,
- $$\langle \nabla v, \Sigma ^\star _x(t,x,v) \rangle \le 0$$.

Then for all $x \in M$ and $0<t \le T$ we have

11.16 $$\begin{aligned} |\nabla v|^s (x,t) \le e^{s(\textsf{k}+a)t} \left\{ \max _{M} \left[ |\nabla v|^s + \Gamma (v) \right] _{t=0} - \Gamma (v(x,t)) \right\} . \end{aligned}$$

### Proof

Using (11.12) and the assumptions on $\Gamma$ and $\Sigma$ in the statement of the corollary, we can write for ${{\textsf{H}}} = {{\textsf{H}}}^s_\zeta [v]= \zeta (t)|\nabla v|^s + \Gamma (v)$

11.17 $$\begin{aligned} {{\mathscr {L}}}&({{\textsf{H}}}) - \frac{2p^2v^{\frac{1}{1-2p}}}{2p-1} \langle \nabla v , \nabla {{\textsf{H}}} \rangle \nonumber \\ \le&~[\zeta '(t)+ s {\textsf{k}} \zeta (t) + s \zeta (t) \Sigma _v^\star (t,x,v)] |\nabla v|^s +sp \zeta (t) \frac{(p-1)^2(m-1)-1}{(2p-1)^2} v^{\frac{2p}{1-2p}} |\nabla v|^{s+2} \nonumber \\&+ s \zeta (t) \langle \nabla v, \Sigma ^\star _x(t,x,v) \rangle |\nabla v|^{s-2} + \Gamma '(v) \Sigma ^\star (t,x,v) -p v^{\frac{1}{1-2p}} [\Gamma '(v)+v\Gamma ''(v)] |\nabla v|^2 \nonumber \\ \le&~[\zeta '(t)+ s ({\textsf{k}} + a) \zeta (t)] |\nabla v|^s +\zeta (t) \frac{s p[(p-1)^2(m-1)-1]}{(2p-1)^2} v^{\frac{2p}{1-2p}} |\nabla v|^{s+2}. \end{aligned}$$

The function $\zeta (t) = e^{-s(\textsf{k}+a)t}$ is non-negative, smooth and satisfies $\zeta '+s(\textsf{k}+a)\zeta =0$. Thus substituting in (11.17) and noting that $(p-1)^2(m-1) \le 1$ as a result of $p_0<p<1$ we conclude that ${{\mathscr {L}}}({\mathsf H}) - 2p^2 v^{1/(1-2p)}/(2p-1) \langle \nabla v, \nabla {{\textsf{H}}} \rangle \le 0$. The assertion is now a consequence of the weak maximum principle. $\square$

### Corollary 11.4

Let $(M,g,e^{-f} dv_g)$ be a smooth metric measure space with *M* closed. Let *u* be a positive smooth solution to (1.1) where $p_0(m)<p <1$ and set $v =u^{p-1/2}$. Assume the metric and potential satisfy the super flow (11.1) with $\textsf{k} \ge 0$. Moreover suppose the following conditions hold:

- $$\Sigma ^\star (t,x,v) \le 0$$,
- $$\Sigma ^\star _v(t,x,v) \le 0$$,
- $$\langle \nabla v, \Sigma ^\star _x(t,x,v) \rangle \le 0$$.

Then for $x \in M$ and $0<t \le T$ we have

11.18 $$\begin{aligned} \frac{t |\nabla v(x,t)|^2}{1+2{{\textsf{k}}}t} \le \frac{(2p-1)^2}{8p^3} \left[ \max _M v^\frac{2p}{2p-1}(x,0) - v^\frac{2p}{2p-1}(x,t) \right] . \end{aligned}$$

### Proof

For ${{\textsf{H}}}[v] = \zeta (t)|\nabla v|^2 + c v^\frac{2p}{2p-1}$ where $\Gamma (v)=cv^\frac{2p}{2p-1}$, we have from Lemma 11.2,

11.19 $$\begin{aligned} {{\mathscr {L}}}&({{\textsf{H}}}) - \frac{2p^2v^{\frac{1}{1-2p}}}{2p-1} \langle \nabla v , \nabla {{\textsf{H}}} \rangle \nonumber \\ \le&~[\zeta '(t)+ 2 {\textsf{k}} \zeta (t) + 2 \zeta (t) \Sigma _v^\star (t,x,v)] |\nabla v|^2 +2p \zeta (t) \frac{(p-1)^2(m-1)-1}{(2p-1)^2} v^{\frac{2p}{1-2p}} |\nabla v|^4 \nonumber \\&+ 2\zeta (t) \langle \nabla v, \Sigma ^\star _x(t,x,v) \rangle + \frac{2pc}{2p-1} v^\frac{1}{2p-1} \Sigma ^\star (t,x,v) - \frac{8p^3c}{(2p-1)^2} |\nabla v|^2. \end{aligned}$$

Recalling the assumptions in the corollary and using $c=(2p-1)^2/(8p^3)$ then gives

$$\begin{aligned} \mathrm{RHS \,} (11.19) \le [\zeta '(t) + 2\textsf{k} \zeta (t) -1] |\nabla v|^2 + \frac{(p-1)^2(m-1)-1}{2p-1} \zeta (t) v^\frac{2p}{1-2p} |\nabla v|^4. \end{aligned}$$

Now taking $\zeta (t) = t/(1+2\textsf{k} t)$ gives $(\zeta ' + 2 \textsf{k} \zeta -1) \le 0$. Therefore since $p_0<p<1$ we have ${{\mathscr {L}}}({{\textsf{H}}}) - [2p^2v^{1/(1-2p)}/(2p-1)] \langle \nabla v, \nabla {{\textsf{H}}} \rangle \le 0$. The conclusion now follows by an application of the weak maximum principle. $\square$

### Remark 11.5

In both corollaries above we can admit the left endpoint of the *p* interval, i.e., $p=p_0(m)$ provided that the strict inequality $p>1/2$ is observed. This specifically means that for $m>5$ where $p_0(m) = 1-1/\sqrt{m-1}>1/2$ we can extend the range to $p_0(m) \le p <1$.

## Data Availability

Data sharing is not applicable to this article as no datasets were generated or analysed during this study.

## Footnotes

1 By the static case we mean the case where the metric tensor *g* and potential *f* are time *independent* which naturally is a very special case.

2 The Riemannian case corresponding to $d\mu =dv_g$, ${{\mathscr {R}ic}}_f^m(g)={{\mathscr {R}}ic}(g)$, $\Delta _f=\Delta$ and $m=n$ (with $n \ge 2$ integer) is where these terminologies on exponents are borrowed from. Here the critical exponent $p_c$ is also known as the Aronson-Bénilan exponent [4].

3 There is another important exponent $$p=1-1/[\sqrt{2m} -1]$$ that lies between $$1-1/\sqrt{m-1}$$ and $$1-2/m $$ and is intimately linked with Theorem 2.1. We do not discuss this explicitly here but it lurks in the background in the proof of Theorem 2.1 and formally appears in Corollary 6.4 (for $$s=2$$) and Remark 6.3 and again in Remark 6.6.
